# Epidemiology of Leptospirosis in Africa: A Systematic Review of a Neglected Zoonosis and a Paradigm for ‘One Health’ in Africa

**DOI:** 10.1371/journal.pntd.0003899

**Published:** 2015-09-14

**Authors:** Kathryn J. Allan, Holly M. Biggs, Jo E. B. Halliday, Rudovick R. Kazwala, Venance P. Maro, Sarah Cleaveland, John A. Crump

**Affiliations:** 1 The Boyd Orr Centre for Population and Ecosystem Health, Institute of Biodiversity, Animal Health and Comparative Medicine, University of Glasgow, Glasgow, United Kingdom; 2 Division of Infectious Diseases and International Health, Department of Medicine, Duke University Medical Center, Durham, North Carolina, United States of America; 3 Sokoine University of Agriculture, Morogoro, Tanzania; 4 Kilimanjaro Christian Medical Centre, Moshi, Tanzania; 5 Kilimanjaro Christian Medical University College, Tumaini University, Moshi, Tanzania; 6 Duke Global Health Institute, Duke University, Durham, North Carolina, United States of America; 7 Centre for International Health, University of Otago, Dunedin, New Zealand; Swiss Tropical and Public Health Institute, SWITZERLAND

## Abstract

**Background:**

Leptospirosis is an important but neglected bacterial zoonosis that has been largely overlooked in Africa. In this systematic review, we aimed to summarise and compare current knowledge of: (1) the geographic distribution, prevalence, incidence and diversity of acute human leptospirosis in Africa; and (2) the geographic distribution, host range, prevalence and diversity of *Leptospira* spp. infection in animal hosts in Africa.

**Methods:**

Following Preferred Reporting Items for Systematic Reviews and Meta-analyses (PRISMA) guidelines, we searched for studies that described (1) acute human leptospirosis and (2) pathogenic *Leptospira* spp. infection in animals. We performed a literature search using eight international and regional databases for English and non-English articles published between January 1930 to October 2014 that met out pre-defined inclusion criteria and strict case definitions.

**Results and Discussion:**

We identified 97 studies that described acute human leptospirosis (n = 46) or animal *Leptospira* infection (n = 51) in 26 African countries. The prevalence of acute human leptospirosis ranged from 2 3% to 19 8% (n = 11) in hospital patients with febrile illness. Incidence estimates were largely restricted to the Indian Ocean islands (3 to 101 cases per 100,000 per year (n = 6)). Data from Tanzania indicate that human disease incidence is also high in mainland Africa (75 to 102 cases per 100,000 per year). Three major species (*Leptospira borgpetersenii*, *L*. *interrogans* and *L*. *kirschneri*) are predominant in reports from Africa and isolates from a diverse range of serogroups have been reported in human and animal infections. Cattle appear to be important hosts of a large number of *Leptospira* serogroups in Africa, but few data are available to allow comparison of *Leptospira* infection in linked human and animal populations. We advocate a ‘One Health’ approach to promote multidisciplinary research efforts to improve understanding of the animal to human transmission of leptospirosis on the African continent.

## Author Summary

Leptospirosis is an important bacterial zoonosis that affects people and animals worldwide. It is common in tropical areas where people and animals live in close contact, but the disease has been widely neglected in Africa. In this study we aimed to demonstrate the extent of leptospirosis in Africa and describe the diversity of the causative agent *Leptospira* spp. in human and animal infections across the continent. Through a systematic literature review, we identified 97 studies from 26 African countries that described human disease or animal infection and met inclusion criteria. Leptospirosis was the cause of illness in 2 3% to 19 8% of hospital patients with a fever. Where population-level data were available, leptospirosis was estimated to affect 3 to 102 people per 100,000 every year. A variety of animal hosts of *Leptospira* spp. were identified. Cattle were reported as carriers of a variety of serological types of *Leptospira* spp. infection. The role of cattle and many other different animal hosts in human disease transmission remains unclear. Our review demonstrates that leptospirosis is a substantial cause of human illness in Africa, and we recommend integration of human and animal studies in the future to help us understand the epidemiology of leptospirosis on this continent.

## Introduction

Endemic zoonotic diseases affect impoverished and developing communities worldwide but are frequently overshadowed in public and clinician awareness by high profile infections such as malaria and HIV/AIDS [1, 2]. In Africa, zoonotic infections are both directly responsible for human illness and death and indirectly impact human well-being as a result of reduced livestock productivity and food security [3–5]. However, bacterial zoonoses including leptospirosis remain under-diagnosed and under-reported in Africa, and as a result are overlooked as public health priorities [1, 2, 6].

Leptospirosis is one of the most common and widespread zoonotic infections in the world and is recognised as a neglected disease by the World Health Organisation (WHO) [7]. Human leptospirosis is caused by infection with pathogenic strains of *Leptospira* spp. bacteria [8, 9]. More than 250 pathogenic *Leptospira* serovars are known to exist worldwide, which are classified into 25 serogroups on the basis of their serological phenotype [10, 11]. Recent species determination by DNA homology has identified 13 pathogenic *Leptospira* spp., and seven of these (*L*. *interrogans*, *L*. *borgpetersenii*, *L*. *santarosai*, *L*. *noguchii*, *L*. *weilli*, *L*. *kirschneri* and *L*. *alexanderi*) are considered as the foremost agents of human and animal disease [10, 12]. Both serological and DNA-based classification systems are currently in use for clinical diagnosis and in understanding the pathogenesis and epidemiology of the disease [11, 13, 14].

A wide range of animals can carry pathogenic *Leptospira* bacteria and act as a source of infection [8, 11]. *Leptospira* serovars often demonstrate a degree of animal host preference and some common relationships between serovars and their hosts are reported [9, 15]. Following infection, the bacteria colonise the renal tubules and urogenital tract and are shed in the urine of infected animals. Animal species may be asymptomatic carriers of infection (maintenance hosts) or develop clinical disease (accidental hosts) depending on the infecting serovar [11, 16]. In food producing animals, cattle and pigs are relatively susceptible to clinical infection resulting in production losses including reduced milk yield, reproductive failure and abortions [16, 17].

In people, disease occurs through direct or indirect contact with infected urine from an animal host [8, 9, 15]. Good knowledge of *Leptospira* serovars circulating in local animal populations is important to determine sources and transmission routes for human infection [8]. In the early stages, human leptospirosis manifests most commonly as a non-specific febrile illness that is hard to distinguish from other aetiologies of febrile disease particularly in tropical areas [11, 18, 19]. Infection can result in severe secondary sequelae including renal failure and pulmonary haemorrhagic syndrome, and a case fatality ratio of up to 50% has been reported in complicated cases [15, 19].

Leptospirosis is particularly common in the tropical areas where people and animals live in close contact, and warm and humid conditions favour environmental survival and transmission of the pathogen [8, 9]. In South-East Asia and South America, leptospirosis is recognised as an important cause of renal failure and febrile disease [18–22]. However, despite its global importance, large gaps persist in our knowledge of the burden and epidemiology of leptospirosis in Africa. Reports from the WHO Leptospirosis Epidemiology Reference Group (LERG) indicate that leptospirosis incidence may be high in Africa, but also highlight the lack of available data [7, 23]. Although reported seroprevalence data demonstrates widespread exposure to *Leptospira* spp. in humans and animals in Africa, [24] little is known about the extent of human disease or the epidemiology of *Leptospira* infection in different animal species in Africa.

To tackle these gaps in current understanding and awareness of human and animal *Leptospira* infection in Africa, we performed a systematic review of peer-reviewed and grey literature following the Preferred Reporting Items for Systematic Reviews and Meta-analyses (PRISMA) guidelines [25]. Our aims were summarise and compare: (1) current knowledge of the geographic distribution, prevalence, incidence and diversity of acute human leptospirosis in Africa; and (2) the geographic distribution, host range, prevalence and diversity of *Leptospira* spp. infection in animal hosts in Africa.

## Methods

### Search strategy

A detailed protocol for this study can be found in the supplementary material (S1 File). Following the PRISMA guidelines and checklist (S1 Checklist) references for this review were identified through searches of eight international and regional databases (Table 1) using the search string ‘Leptospirosis’ OR ‘*Leptospira*’ and ‘Africa*’ for articles published between January 1930 and October 2014 inclusively. Additional articles for inclusion were identified by bibliography hand searches of relevant articles [26].

**Table 1:** Full search strategies for database searches (in alphabetical order).

Database	Publication Date Limits	Search Strategies
Africa-Index Medicus (World Health Organization Global Health Library)	January 1930-October 2014	(Leptospirosis OR *Leptospira*)
Africa-Wide:NiPAD (now EBSCO Host Africa Wide Information)	January 1930-October 2014	(SU (leptospirosis OR leptospira) OR TX (leptospirosis OR leptospira)) AND (AB Africa* OR GE africa OR SU africa OR TI Africa* OR KW africa)
BIOSIS Previews	January 1930-October 2014	Search 1: Topic = (leptospirosis) OR Topic = (leptospira) OR title = (leptospirosis) OR title = (leptospira); Search 2: topic = (Africa*) OR title = (Africa*); Search 3: Combine Search 1 AND Search 2
CAB International: CAB abstracts and Global Health	January 1930-October 2014	Search 1: Topic = (leptospirosis) OR Topic = (leptospira) OR title = (leptospirosis) OR title = (leptospira); Search 2: topic = (Africa*) OR title = (Africa*); Search 3: Combine Search 1 AND Search 2
Embase (Ovid; including Embase Classic and Embase)	January 1947-October 2014	((leptospirosis [sh] OR leptospira [sh] OR leptospirosis [tw] OR leptospira [tw]) AND (africa*[sh] OR africa*[tw]))
Pubmed	January 1930-October 2014	((leptospirosis[mesh] OR leptospirosis[Text Word] OR leptospira[Text Word] OR leptospira[mesh)) AND (africa[mesh] OR africa*[Text Word]))
Web of Science Core Collection	January 1930-October 2014	Search 1: Topic = (leptospirosis) OR Topic = (leptospira) OR title = (leptospirosis) OR title = (leptospira); Search 2: topic = (Africa*) OR title = (Africa*); Search 3: Combine Search 1 AND Search 2
Zoological Record	January 1930-October 2014	Search 1: Topic = (leptospirosis) OR Topic = (leptospira) OR title = (leptospirosis) OR title = (leptospira); Search 2: topic = (Africa*) OR title = (Africa*); Search 3: Combine Search 1 AND Search 2

### Study selection and criteria

Abstracts and titles were compiled in EndNote (Thomson Reuters, Philadelphia, PA, USA) and reviewed independently by two researchers (KJA, HMB) to determine whether each article met pre-determined abstract inclusion and exclusion criteria (S1 File). A third researcher (JEBH) served as a tiebreaker for any discordant decisions. Citations were included if they presented data on human or animal *Leptospira* spp. infection from any country within the United Nations (UN) definition of Africa [27]. We excluded abstracts that did not refer to original human or animal leptospirosis research data, or did not describe naturally occurring cases of leptospirosis in human or animal populations. We included case reports but excluded reports of returned travellers because of potential uncertainty around the specific location where infection was acquired.

Articles classified as eligible for inclusion were retrieved in full text format and assessed against pre-defined case definitions (Table 2) of human acute leptospirosis and carrier animal status agreed upon by three authors (KJA, HMB, JEBH). Rigorous diagnostic criteria were specified in accordance with WHO and international reference laboratory guidelines (Table 2) [7, 11, 16]. Serological diagnostics were not included in the case definition for carrier animals because of the inability to differentiate between previous exposure and current infection status. We also excluded articles describing studies that used laboratory animal inoculations as a diagnostic test for leptospirosis because of concerns over the risk of false positive results as a consequence of pre-existing infection in experimental animal colonies, diagnostic sensitivity and cross-contamination [16]. Full text articles were reviewed by two authors (KJA, HMB) and were excluded if they failed to meet case definitions, if results from the same cohort were presented more comprehensively in another eligible article, or if insufficient information was provided in the study methodology to determine whether the case definitions were met. Non-English language articles identified for full text review (n = 97) included French language articles translated by KJA with assistance from a native language speaker (n = 83); German language articles translated by a native language speaker (n = 7); Italian articles translated by a native language speaker (n = 4); Afrikaans (n = 2) and Dutch language articles (n = 1), which were translated using online translation software with support from a Dutch language speaker [28].

**Table 2:** Case definitions for study inclusion: Acute human leptospirosis and confirmed animal carrier hosts.

**Human acute disease case definition, confirmed**
Compatible acute illness, plus ≥1 of the following:
≥ 4 fold rise in Microscopic Agglutination Test (MAT) titre between acute and convalescent serum
Culture* and isolation of pathogenic *Leptospira* spp. from blood, urine, CSF or tissues
Pathogenic *Leptospira* spp. DNA detected by Polymerase Chain Reaction (PCR) from blood/blood derivatives, urine, cerebrospinal fluid, or tissues
Detection of *Leptospira* spp. in tissue by immunohistochemical techniques
**Human acute disease case definition, probable**
Compatible acute illness, plus ≥1 of the following:
MAT titre ≥1:400 in single or paired serum samples
Presence of IgM antibodies by enzyme-linked immunosorbent assay (ELISA) or dipstick
Presence of IgM or a fourfold increase in IFA antibody titre in acute and convalescent serum samples
**Animal carrier case definition, confirmed**
Clinical signs present or absent, plus ≥ 1 of the following:
Culture* and isolation of pathogenic *Leptospira* spp. from a normally sterile site
Pathogenic *Leptospira* spp. DNA detected by PCR or real-time PCR (qPCR) from a normally sterile site
Typing of previously isolated strain
Detection of *Leptospira* spp. in clinical specimens by immunohistochemical techniques

Footnotes

* Culture in any of the following media: Ellinghausen-McCullough-Johnson-Harris (+/- 5’Fluorouracil), Fletcher, Korthoff, Stuart, Vervoot or Noguchi culture media.

### Data extraction and synthesis

Two reviewers (KJA, HMB) independently extracted pre-determined qualitative and quantitative data from each included article. Data on infection prevalence and incidence for comparable studies (i.e. similar study inclusion criteria and diagnostic methodologies) were compiled, and ranges were presented by study type (human studies), location or host species (animal studies) if three or more citations reporting comparable data were identified. Data on serological and genetic typing of leptospiral isolates from people and animals were compiled and summarised by country and by animal species. Additional data on serogroup and genetic species of reported serovars was obtained from the Leptospirosis Library, maintained by the Leptospirosis Reference Centre, Royal Tropical Institute (KIT), Netherlands [29].

### Critical assessment of methodological quality and bias

The risk of bias in included studies such as selection or reporting bias was assessed following the Cochrane guidelines for systematic reviews of medical interventions [30]. Full text study validity and methodological quality was assessed by comparison to pre-determined case definition criteria to control for heterogeneity in study design and diagnostic methodology (Table 2). Studies classified as high-risk for bias were not included in quantitative analysis of leptospirosis prevalence and incidence data.

## Results

**Fig 1:**
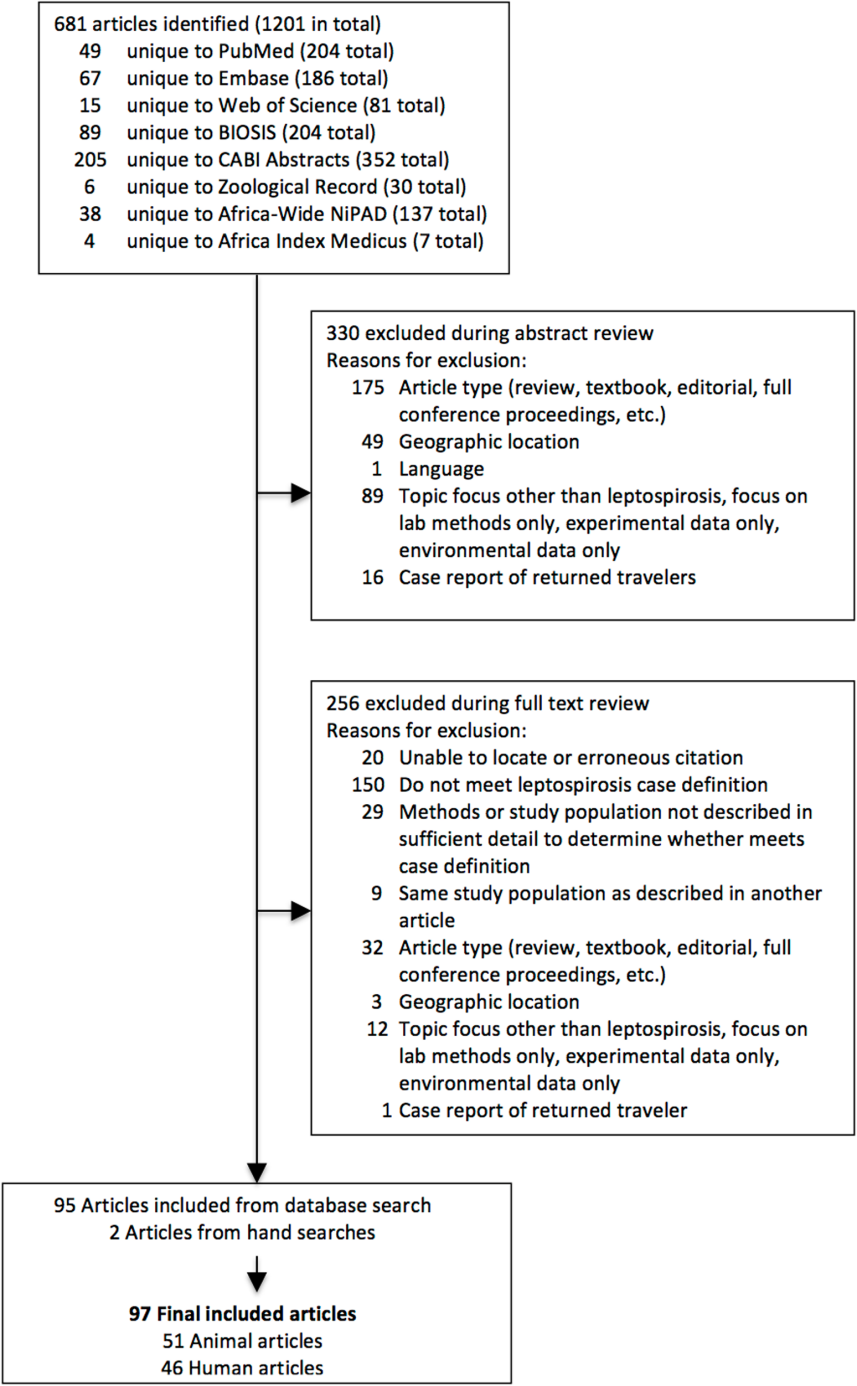
PRISMA flowchart. Selection of eligible articles for study inclusion.

**Fig 2:**
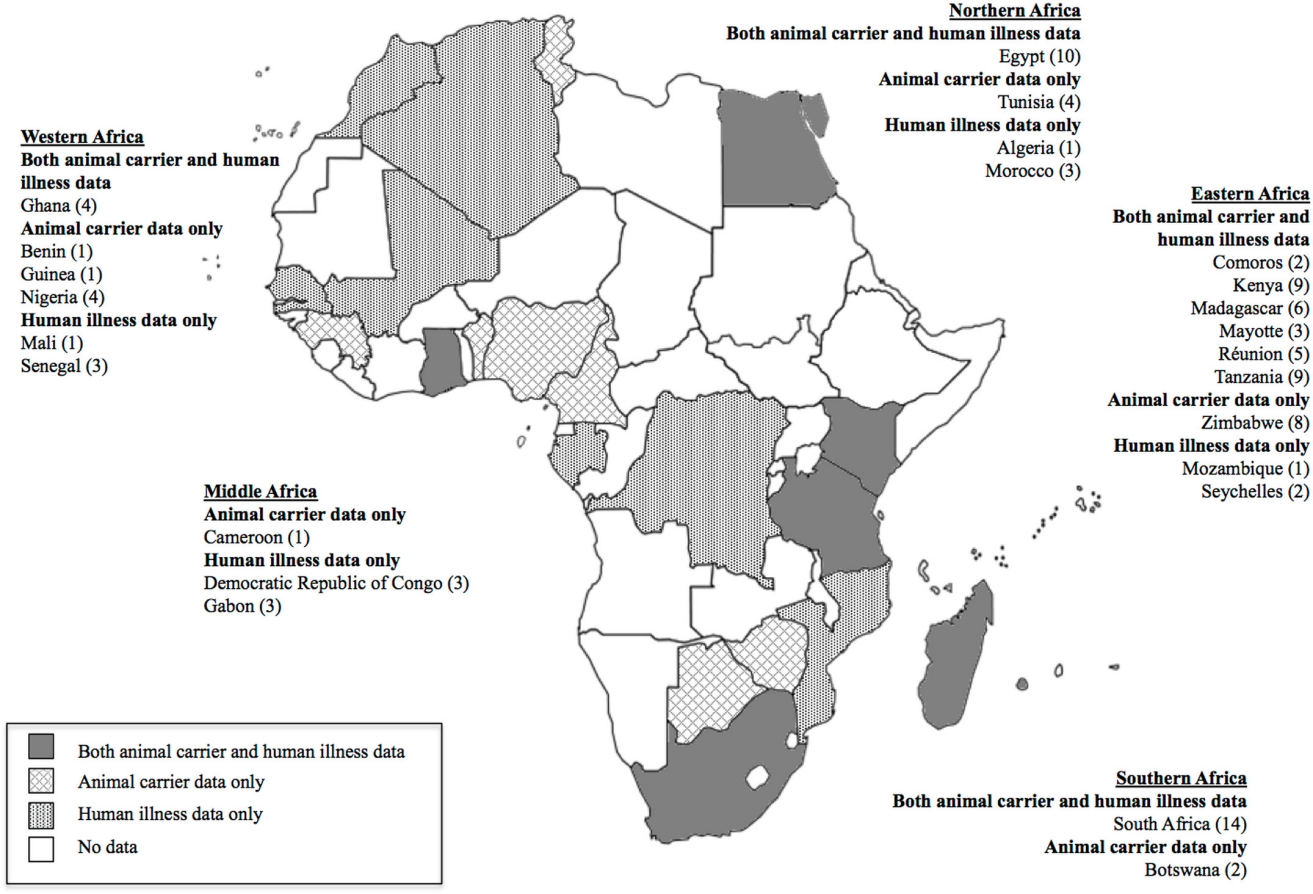
Geographic distribution of acute human leptospirosis and confirmed animal *Leptospira* spp. infection in Africa.

Our searches yielded 681 unique articles from a total of 1201 abstracts identified by database searches. Data can be accessed through: http://dx.doi.org/10.5525/gla.researchdata.191 . After abstract and full text review, 95 citations published between 1956 and 2014 were eligible for inclusion. Hand searches identified two additional articles that met inclusion criteria but were not identified in the original database search. Reasons for full-text exclusion are detailed in Fig 1. In total we included 97 articles that described human or animal studies conducted in 26 (44.8%) of 58 countries included in the UN macro-geographical definition of the African continent (Fig 2) [27]. Major risks of bias identified in eligible studies were selection bias, attrition bias in studies that relied on paired serology (MAT) for confirmatory diagnosis, and reporting bias, as descriptions of diagnostic methodology and results were often incomplete.

### Acute human leptospirosis studies

Acute human leptospirosis was reported in 46 eligible studies from 18 African countries (Fig 2) [31–76]. South Africa was the most frequently represented country with a total of six articles [43, 47, 54, 57, 65, 71], followed by Egypt [45, 55, 56, 58, 59] and Kenya [31, 37–39, 42] with five included articles. Twenty-one articles described acute human leptospirosis in hospital or health centre-based cohort studies (Table 3). Five articles described data from passive population-based surveillance [35, 41, 64, 70, 73], and two articles described active case-finding in the setting of an outbreak of acute febrile illness [31, 72]. Non-specific febrile illness was the most common clinical criteria described for cohort or surveillance study inclusion. Jaundice was stated as a primary inclusion criterion in three hospital-based cohort studies [44, 61, 66]. Haemoglobinuria was stated as the only inclusion criterion in one study conducted in the Democratic Republic of the Congo (DRC) [40].

**Table 3:** Summary of eligible cohort and surveillance studies reporting human acute leptospirosis in Africa, 1930–2014.

Citation	Study year(s)	Country	Setting and study design	Inclusion and exclusion criteria	Diagnostic tests	Number enrolled	Total number of eligible cases* (%)	No. of eligible cases: confirmed & probable *
Van Riel et al[69]	1952–54	Democratic Republic of Congo (DRC)	Hospital; retrospective cohort	Clinical suspicion of leptospirosis	Culture (blood) in Vervoort-Korthoff media; Agglutination-lysis (MAT)	45	27 (60 0%)	5 confirmed, 22 probable
Kolochine-Erber & Brygoo[49]	1954–55	Madagascar	Undefined; prospective cohort	Clinical suspicion of leptospirosis	Agglutination-lysis (MAT)	40	1 (2 5%)	1 probable
Forrester et al[42]	1961–62	Kenya	Hospital; prospective cohort	Febrile illness unexplained by malaria, dysentery or pneumonia.	MAT	67	6 (9 0%)	All probable
Payet et al[61]	1964–65	Senegal	Hospital; prospective cohort	Clinical suspicion of leptospirosis; mostly defined by jaundice	Agglutination-lysis (MAT)	53	3 (5 7%)	2 confirmed, 1 probable
Silverie et al[67]	1966–67	Madagascar	Undefined; prospective cohort	Clinical suspicion of leptospirosis	Agglutination-lysis (MAT)	65	7 (10 8%)	All probable
De Geus et al[37]	1967	Kenya	Hospital and health centre; prospective cohort	Febrile illness (temperature ≥ 38°C) without obvious cause; negative malaria smear or no response to anti-malarial treatment	Culture (blood) in Fletcher’s and Cox’s media; MAT	39	7 (17 9%)	6 confirmed, 1 probable
Sankale et al[66]	1967–72	Senegal	Hospital; retrospective cohort	Inpatients with serum samples tested for leptospirosis	Serum agglutination (MAT)	134	3 (2 2%)	All confirmed
De Geus et al[39]	1968–69	Kenya	Hospital outpatient department and health centre; prospective cohort	Febrile illness (temperature ≥ 38.3°C) without obvious cause; negative malaria smear or no response to anti-malarial treatment ^a^	Culture (blood) in Fletcher’s media; MAT	91	10 (11 0%)	All confirmed
De Geus et al[38]	1969	Kenya	Hospital & outpatient department; prospective cohort & case-finding survey ^b^	Febrile illness (temperature ≥ 38.3°C) without obvious cause; negative malaria smear or no response to anti-malarial treatment	Culture (blood) in Fletcher’s media; MAT ^c^	281	9 (3 2%)	All confirmed
Kinebuchi et al[46]	NA	Ghana	Hospital; prospective cohort	Clinical suspicion of leptospirosis, mostly defined by hepatitis or jaundice	Culture (blood) in Korthof’s media; MAT	99	13 (13 1%)	7 confirmed,6 probable
Hogerzeil et al[44]	1981–82	Ghana	Hospital outpatient department; prospective cohort	Group 1: Fever without obvious cause and/or any of the following; jaundice, muscle pains, meningism, conjunctival injection, albuminuria; negative malaria smear	Culture (blood and urine) in Fletcher’s or Ellinghausen-McCullough-Johnson-Harris	Group 1: 88	Group 1: 4 (4 5%)	Group 1: 3 confirmed; 1 probable
				Group 2: Jaundice		Group 2: 102	Group 2: 2 (2 0%)	Group 2: All confirmed
Delacollette et al[40]	1985–86	DRC	Hospital; prospective cohort	Inpatients with black or red urine with confirmed haemoglobinuria	ELISA (unspecified)	38	1 (2 6%)	All probable
Pinn[63]	1988–90	Seychelles	Hospital; prospective cohort	Inpatients with clinical diagnosis of leptospirosis ^d^	IgM ELISA	80	58 (72 5%)	All probable
Collares-Pereira et al[36]	1993	Mozambique	Hospital outpatient department; prospective cohort	Outpatients aged 18–50 years with acute febrile illness without obvious cause; negative malaria smear.	MAT	43	1 (2 3%)	1 probable
Yersin et al[70]	1995–96	Seychelles	Nationwide health care providers; Prospective population-based surveillance	Fever or any of the following without obvious cause: myalgia, liver tenderness, jaundice, acute renal failure, bleeding tendency, radiographic lung infiltrates, or meningism	MAT; PCR (*rrs*)	125	75 (60 0%)	All confirmed
Desvars et al[41]	1998–2008	Réunion	Hospital; retrospective population-based surveillance	Cases voluntarily reported to Centre National de References de Leptospiroses (Paris, France)	Culture (blood), media not specified; MAT; PCR (target not specified)	NA	613 cases	All probable**
Ismail et al[45]	1999–2003	Egypt	Hospital; retrospective cohort	Group 1: fever (temperature ≥38°C) for ≥3 days in the absence of diarrhoea, pneumonia, typhoid fever, brucellosis or established fever of unknown origin.	IgM ELISA; MAT	Group 1:886 ^e^	Group 1: 141 (15 9%)	All probable**
				Group 2: acute hepatitis defined as signs of acute jaundice.		Group 2: 392 ^f^	Group 2: 63 (16 1%)	
Renault et al[64]	2004–08	Réunion	Hospital; retrospective population-based surveillance	Hospitalised cases of leptospirosis cases in Réunion reported to the Regional Directorate for Health and Social Affairs/Regional Health Agency of the Indian Ocean.	Confirmed cases: Culture (not specified), MAT or PCR (target not specified)	240	160 (66 7%)	All probable**
					Possible cases: IgM ELISA; MAT titre ≥ 1:50			
Pages et al[73]	2004–12	Réunion	Population-based surveillance	Confirmed or probable cases of leptospirosis in Réunion residents reported to the health watch platform of the French Regional Health Agency for the Indian Ocean.	Confirmed cases: Culture (not specified), MAT or PCR (target not specified)	NA	405 cases	All probable**
					Possible cases: IgM ELISA.			
Ari et al[31]	2005	Kenya	Community; prospective case-finding^g^	Community members with new onset febrile illness (temperature not defined) or joint pains	IgM ELISA	12	3 (25 0%)	All probable
Bertherat et al[72]	2005	DRC	Community; retrospective case finding	Acute & convalescent patients with respiratory disease in a mining camp	MAT	82	8 (9 8%)	All probable
Parker et al[58]	2005–2006	Egypt	Hospital; prospective cohort	Fever ≥ 2 days or admission temperature ≥38.5°C, aged ≥ 4 years without obvious cause of fever, such as diarrhoea, pneumonia, or clinical diagnosis of typhoid fever or brucellosis.	Culture (blood) in EMJH; MAT; PCR; IgM ELISA	981	194 (19 8%)	45 confirmed; 149 probable
Parker et al[59]	2005–2006	Egypt	Hospital; prospective cohort	Fever ≥ 2 days or admission temperature ≥38.5°C, aged ≥ 4 years without obvious cause of fever; with laboratory evidence of co-infection with *Leptospira*, *Rickettsia typhi*, *Brucella*, or *Salmonella enterica* serogroup Typhi	Culture (blood) in EMJH; MAT; PCR (*lig*A)	187 ^h^	152 (81 3%)	All confirmed
Murray et al[55, 56]	2005–2007	Egypt	Hospital; prospective cohort	Fever; aged ≥ 4 years without obvious cause of fever, such as diarrhoea, pneumonia, or clinical diagnosis of typhoid fever or brucellosis.	Culture (blood) in EMJH media; MAT; PCR (*lig*A)	2,441	98 (4 0%)	All probable**
Tagoe et al[68]	NA	Ghana	Hospital; prospective cohort	Fever ≥ 2 days and temperature ≥38.0°C; aged ≥ 4 years without obvious cause of fever	IgM ELISA; MAT	166	13 (7 8%)	All probable
Biggs et al[33, 74]	2007–08	Tanzania	Hospital; prospective cohort	Inpatients aged ≥13 years with fever (≥38.0°C oral) or inpatients aged 2 months to 12 years with history of fever within 48 hours or admission temperature ≥37.5ºC axillary ≥38.0ºC rectal.	MAT	831	70 (8 4%);	40 confirmed, 30 probable
Bourhy et al[35]	2007–08	Mayotte	Undefined; prospective cohort	Fever (temperature ≥38°C) for ≤7days and headache and/or myalgia	Culture (blood) in EMJH media; PCR (*rrs*)	388	53 (13 7%),	All confirmed
Bourhy et al[34]	2007–2010	Mayotte ^k^	Undefined; population-based surveillance	Patients for which a blood sample was submitted for leptospirosis diagnosis to the Hospital Centre of Mayotte	Culture (blood) in EMJH media; PCR (*lbf1*, *lipL32*, *rrs*)	2,523	198 (7 8%)	All confirmed

Footnotes

*Figures reported here are based on the number of reported acute leptospirosis cases that met our review case definitions (see Table 1 for case definitions) and therefore may vary from the values reported in the original citations.

** All cases met probable case definitions. An unspecified proportion of positive cases also met the case definition for confirmed cases but exact numbers could not be determined from the available data.

^a^ Patients who refused hospital admission were not investigated.

^b^ Methods describe a change to a case-finding survey partway through the study, but full details not available

^c^ MAT performed in a subset of participants only

^d^ Clinical diagnosis defined as ≥3 of the following: headache or fever (temperature not defined), evidence of liver inflammation (defined as jaundice, tender liver, and/or abnormal liver function tests), evidence of renal inflammation (haematuria and/or abnormal renal function), or evidence of muscle inflammation (tenderness and/or elevated creatine phosphokinase)

^e^ All tested negative for *Salmonella enterica* serovar Typhi, *Brucella* spp., and *Rickettsia* spp.

^f^ All tested negative for Hepatitis A, B, and C.

^g^ In setting of outbreak of acute febrile illness in a well-defined population

^h^ 187 patients were diagnosed with selected co-infections out of a total cohort of 1510 patients with non-specific febrile illness.

ϖ Taken ≥ 9 days of onset of illness

^k^ Also report two imported cases from Comoros and Madagascar respectively

### Diagnostic methodologies for human studies

The majority of studies (n = 41/46) used microscopic agglutination test (MAT) as a primary method to diagnose human cases of acute leptospirosis. IgM enzyme linked immunosorbent assay (ELISA) testing was the only diagnostic method used in three studies [31, 40, 63], but was more commonly used as part of a multi-faceted diagnostic approach (n = 6/46) [44, 45, 58, 64, 68, 73]. Fifteen (32 6%) of 46 eligible human studies demonstrated leptospirosis infection by blood culture in combination with serological diagnostics [34, 35, 37–39, 41, 44, 54–56, 58, 59, 64, 69], and nine (19 5%) studies also used PCR detection as well as culture and serology [34, 35, 41, 56, 58, 59, 64, 70, 73]. Genetic targets for diagnostic PCR assays included *lbf1*,[34, 35] *lipL32* [34, 35], *rrs* [34, 35, 70], and *ligA* [58, 59]. No culture-independent genetic typing of *Leptospira* spp. was reported in any included human studies.

### Human leptospirosis prevalence

Leptospirosis prevalence varied by study design and inclusion criteria (Table 3). In hospital-based prospective cohort studies in mainland Africa that enrolled patients with non-specific febrile illness and used MAT serology for diagnosis of acute leptospirosis with or without adjunct diagnostics, prevalence ranged from 2 3% to 19 8% (n = 11; number of patients: median = 166; range = 39–2441) [33, 36–39, 42, 44, 45, 55, 58, 68]. A hospital-based prospective cohort study of febrile patients in Mayotte that diagnosed acute leptospirosis by PCR and culture without serology reported a prevalence of 13 7% (number of patients = 2523) [34]. In hospital-based cohort studies that used jaundice as the main study enrolment criterion, prevalence of acute leptospirosis ranged from 2 0% to 16 1% (n = 3; number of patients: median = 102; range = 99–392) [44–46]. Acute leptospirosis was also reported in one patient (2 3%) of 38 with haemoglobinuria [40], three patients (25 0%) of 12 involved in an outbreak of acute febrile disease in a pastoralist community in northern Kenya [31], and eight patients (9 8%) of 82 involved in an outbreak of acute pulmonary disease (pneumonia) in a mining camp in DRC [72].

### Human leptospirosis incidence

Incidence estimates were calculated in five population-based surveillance studies [35, 41, 64, 70, 73] and two hospital-based prospective cohort studies [63, 74]. The only estimate of incidence from mainland Africa came from northern Tanzania, where regional incidence of 75 to 102 cases per 100,000 people per year was reported. This estimate was obtained by combining data on leptospirosis prevalence from hospital-based surveillance of febrile disease with multipliers derived from a population-based health-care utilisation survey [74]. For the Indian Ocean islands, incidence estimates were available for the Seychelles where the average annual incidence was estimated as 60 to101 cases per 100,000 [63, 70]; Réunion where the average annual incidence reported in three studies using a variety of data sources ranged from 3 1 to 12 0 cases per 100,000 [41, 64, 73] and Mayotte, where the average annual incidence calculated from cases identified through four years of active hospital-based surveillance between 2007 and 2010 was reported as 25 cases per 100,000 [35].

### Human case reports

Sixteen case reports describing acute leptospirosis in a total of 34 individuals were considered eligible for study inclusion. A wide range of clinical manifestations were reported including febrile illness, jaundice, meningitis, and acute respiratory distress syndrome. Case reports described confirmed or probable acute leptospirosis in patients in South Africa (n = 6) [43, 47, 54, 57, 65, 71], Gabon (n = 3) [48, 62, 76], Morocco (n = 3) [50, 52, 53], Algeria (n = 1) [32], Mali (n = 1) [51], Réunion (n = 1) [75], and Senegal (n = 1) [60]. With the exception of Réunion and Senegal, case reports were the only eligible data on acute human leptospirosis from these countries.

### Animal *Leptospira* infection studies

Naturally occurring *Leptospira* spp. infection in animal hosts was reported by 51 eligible citations describing studies performed in 17 African countries (Fig 2) [77–127]. South Africa [84, 100, 101, 104, 117, 120–122] and Zimbabwe [83, 93–99] were the most frequently represented countries with a total of eight included articles per country, followed by Tanzania with seven articles [106, 110–112, 114–116]. Wild animal surveys were most commonly described (n = 21/51) followed by strain typing of *Leptospira* spp. previously isolated from naturally infected animal hosts (n = 13/51), livestock disease outbreaks (n = 7/51) and abattoir surveys (n = 7/51). Four citations (n = 4/51) reported human leptospirosis outbreaks as the inciting cause for investigations into animal carrier status [86, 109, 117, 123].

### Carrier animal species

*Leptospira* spp. infection was demonstrated in a wide range of animal hosts (S1 Table), including cattle (*Bos* spp.) [85, 87, 89–91, 93–102, 111, 114, 119, 121, 127]; pigs (*Sus scrofa domestica*) [78, 79, 84, 85, 100, 104, 106, 122]; goats (*Capra aegagrus hircus*) [85]; Rusa deer (*Rusa timorensis*) [85]; dogs (*Canis lupis familiaris*) [85, 113, 116]; cats (*Felis catus*) [85, 113, 116]; rodents including the African grass rat (*Arvicanthus niloticus*) [87, 88], African giant pouched rat (*Cricetomys gambianus*) [110, 112], lesser tufted-tailed rat (*Eliurus minor*) [125], fringe-tailed Gerbil (*Gerbilliscus robustus*) [77, 88], rusty-bellied brush-furred rat (*Lophuromus sikapusi*) [109], multimammate mouse (*Mastomys* sp.) [83, 87, 103, 115], house mouse (*Mus musculus*) [80, 81, 83, 85, 118, 120, 124], brown rat (*Rattus norvegicus*) [82, 85, 103, 108, 117, 118, 120, 124], black rat (*Rattus rattus*) [83, 85, 86, 92, 103, 118, 120, 124], South African pouched mouse (*Saccostomys campestris*) [88]; and a range of other free-living mammal species including shrews (*Crocidura* spp. and *Suncus murinus*) [86, 103, 115, 118]; mongoose (*Herpestes ichneumon*, *Mungo mungo* and *Paracynictic selousi)* [80, 105]; Egyptian fox (*Vulpes vulpes niloticus*) [80]; shrew tenrecs (*Microgale cowani*, *Microgale dobsoni*, *Microgale longicaudata*, *Microgale majori*, *Microgale principula*) [125]; streaked tenrecs (*Hemicentetes nigriceps*, *Hemicentetes semispinosus*) [125]; and various bat species (*Chaerephon pusillus*, *Miniopterus gleni*, *Miniopterus goudoti*, *Miniopterus griffithsi*, *Miniopterus griveaudi*, *Miniopterus mahafaliensis*, *Miniopterus majori*, *Miniopterus soroculus*, *Mormopterus francoismoutoui*, *Mormopterus jugularis*, *Mytotis goudoti*, *Otomops madagascariensis*, *Rousettus obliviosus*, *Triaenops furculus*, *Triaenops menamena*) [107, 125]. Studies demonstrating infection in cattle were most common (n = 20/51) followed by pigs (n = 8/51), black rats (n = 8/51), brown rats (n = 7/51) and house mice (n = 7/51).

### Diagnostic methodologies for animal studies

Culture and isolation was the most common detection method for *Leptospira* infection in animal studies (n = 43/51). PCR assays were used to demonstrate *Leptospira* spp. infection in 13 (25 5%) out of 51 studies [85, 86, 92, 103, 105, 107, 115, 118, 120, 123–126]. In three studies, culture and PCR were used in combination to determine infection status [92, 115, 118]. As with human studies, a variety of genetic targets were used in PCR assays to detect pathogenic leptospiral DNA, including *lipL32/hap1*, [85, 86, 118], *secY*, [103], *rrl* [105], and *rrs* [107, 115, 120]. PCR was predominantly used to demonstrate *Leptospira* spp. infection in rodents and wild animal species. Only one study in Réunion also used PCR assays to demonstrate infection in domestic animals [85].

### Prevalence in animal populations

*Leptospira* infection prevalence varied widely by target animal species and diagnostic methodology (S1 Table). Studies that used PCR diagnosis reported higher infection prevalence than studies that relied on *Leptospira* culture and isolation. Overall *Leptospira* infection prevalence reported in black rats tested by PCR ranged from 11 0% to 65 8% (n = 6; number of animals: median = 79, range = 33–141) [85, 86, 92, 103, 118, 124]. In two studies where black rats were tested by both PCR and culture, prevalence was higher by PCR (11 0%, n = 100; and 28.7%, n = 94) than by culture (4 0% and 3 2%) in Egypt [92], and Madagascar respectively [118]. A similar relationship was observed in brown rats, house mice and Asian house shrews tested in Madagascar [118]. Cattle and brown rats were the most common species tested by culture. Prevalence in brown rats ranged from 2 7% to 8 5% by culture (n = 3; number of animals: median = 256, range = 130–919) [82, 108, 117] but was considerably higher in three studies that used PCR to detect infection (10 0% to 4 7%; number of animals: median = 11, range = 10–96) [103, 118, 124]. In four abattoir-based surveillance studies of cattle from Egypt, Nigeria and Zimbabwe [87, 89, 93, 127], renal *Leptospira* spp. carrier status was detected by culture in 1 1% to 10 4% of sampled animals (number of animals: median = 480, range = 74–625), compared to 18 2% (number of animals = 77) in a single PCR-based study from Mayotte [85].

### Serological typing of infecting leptospires in humans and animals

Serological typing of *Leptospira* spp. isolates from patients with acute leptospirosis was described in cohort studies conducted in the DRC [69], Egypt [55, 56], Ghana [44], Kenya [37–39] and Mayotte [34, 35], and in a case report from South Africa [54]. Isolates belonging to 15 serogroups were reported (Table 4). Mini and Icterohaemorrhagiae were the most commonly reported serogroups. Isolates that were equally cross-reactive with representative serovars from more than one serogroup (Mini/Hebdomadis and Pyrogenes/Ballum) were reported by two studies in Mayotte [34, 35]. In animal studies, isolates belonging to 12 serogroups were reported from 33 articles. At least one animal host was identified within Africa for 11 (73 3%) out of the 15 human-infecting serogroups identified in this review (Table 4). However, only six of these serogroups were detected in human and animal populations from the same country. These were serogroup Autumnalis in Kenya [39, 88]; and serogroups Canicola [56, 92, 113], Grippotyphosa [56, 80, 81, 92], Icterohaemorrhagiae [55, 80, 92, 127], Pomona [55, 56, 127] and Pyrogenes [56, 92] in Egypt. Serogroups associated with human febrile illness were frequently isolated from multiple animal hosts. One of the most commonly reported serogroups isolated from patients in Africa, serogroup Icterohaemorrhagiae, was isolated from cattle, brown rats, Egyptian mongoose and an Egyptian fox. Cattle were identified as carrier hosts for the widest range of *Leptospira* serogroups (n = 9) but several other animal species, such as African grass rats and black rats were also identified as carrier hosts for multiple serogroups.

**Table 4:** Serogroups of *Leptospira* isolated from cases of acute human leptospirosis and animal carrier hosts by country.

	Human Studies	Animal Studies	
Serogroup	Country	Host species	Country
**Australis**	Kenya[39]	African grass rat (*Arvicanthus niloticus*)	Nigeria[87]
		Cattle (*Bos* spp.)	Zimbabwe[93]
**Autumnalis**	Kenya[39]	African grass rat (*Arvicanthus niloticus*)	Kenya[88]
**Ballum**	Not reported	African giant pouched rat (*Cricetomys gambianus*)	Tanzania[110, 112]
		African grass rat (*Arvicanthus niloticus*)	Nigeria[87]
		South African pouched mouse (*Saccostomys campestris*)	Kenya[88]
**Bataviae**	Egypt[55, 56]	Cattle (*Bos* spp.)	Zimbabwe[93, 94]
		Rusty-bellied brush-furred rat (*Lophuromys sikapusi*)	Cameroon[109]
**Canicola**	Egypt[56]	Black rat (*Rattus rattus*)	Egypt[92], Madagascar[118]
	Kenya[39]	Brown rat (*Rattus norvegicus*)	Madagascar[118]
	South Africa[54]	Dogs (*Canis lupus familiaris*)	Egypt[113]
		Pigs (*Sus scrofa domesticus*)	South Africa[122]
**Djasiman**	Ghana[44]	Not reported	
**Grippotyphosa**	DRC[69]	Black rat (*Rattus rattus*)	Egypt[92]
	Egypt[56]	Cattle (*Bos* spp.)	Kenya[119] Zimbabwe[93, 97]
	Mayotte [34, 35]	House mouse (*Mus musculus*)	Egypt[80, 81]
**Hebdomadis**	DRC[69]	Cattle (*Bos* spp.)	Zimbabwe[93, 99]
	Kenya[37–39]		
**Icterohaemorrhagiae**	Egypt[55, 56]	Brown rat (*Rattus norvegicus*)	South Africa[117], Tunisia[108]
	Ghana[44]	Cattle (*Bos* spp.)	Egypt[127], Tanzania[114], Zimbabwe[93, 95]
	Kenya[37, 38]	Egyptian fox (*Vulpes vulpes niloticus*)	Egypt[80]
		Egyptian mongoose (*Herpestes ichneumon*)	Egypt[80]
**Mini**	Mayotte[34, 35]	Not reported	
**Pomona**	Egypt[55, 56]	Cattle (*Bos* spp.)	Botswana[102], Egypt[127], South Africa[100, 101], Zimbabwe[93, 97]
	Mayotte[35]	Pigs (*Sus scrofa domesticus*)	South Africa[84, 100, 104]
**Pyrogenes**	Egypt[56]	Black rat (*Rattus rattus*)	Egypt[92]
	Kenya[39]	Cattle (*Bos* spp.)	Nigeria[87, 89–91], Zimbabwe[93, 98]
	Mayotte[34, 35]		
**Sejroe**	Not reported	Black rat (*Rattus rattus*)	Egypt[92]
		Cattle (*Bos* spp.)	South Africa[121], Zimbabwe[93]
**Tarassovi**	DRC[69]	Cattle (*Bos* spp.)	Zimbabwe[93, 96]
		Fringe-tailed gerbil (*Gerbilliscus robustus*)	Kenya[88]
		Pigs (*Sus scrofa domesticus*)	Tunisia[78, 79]
**Wolfii**	Egypt[56]	Not reported	
**Mini/Hebdomadis** *	Mayotte[34, 35]	Not reported	
**Pyrogenes/Ballum** *	Mayotte[35]	Not reported	

Footnotes

* Cross-reactive isolates

### Genetic typing of leptospires in humans and animals

*Leptospira* spp. isolated from human patients with acute leptospirosis belonged to five pathogenic *Leptospira* species (Table 5). *L*. *interrogans* was the most widespread and common species reported in either human or animal studies in Africa. Multiple animal hosts were identified for *L*. *interrogans* as well as the other common species, *L*. *borgpetersenii* and *L*. *kirschneri*, from a variety of countries. The widest diversity in *Leptospira* spp. was reported from two Kenyan studies of acute human leptospirosis, where isolates belonging to five species were identified (*L*. *borgpetersenii*, *L*. *interrogans*, *L*. *kirschneri*, *L*. *noguchii* and *L*. *santarosai*) [38, 39]. However, *L*. *noguchii* and *L*. *santarosai* were not detected in any other studies. Four *Leptospira* species: *L*. *borgpetersenii*, *L*. *borgpetersenii*-like, *L*. *interrogans* and *L*. *kirschneri*; were identified in two human studies on Mayotte, as well as by a concurrent study of black rats performed during the same period [34, 35, 86]. Divergent *Leptospira* spp. described as *L*. *borgpetersenii-*like and *L*. *borgpetersenii* Group B were detected in human and animal studies respectively in Mayotte, and in a study of indigenous small mammals in Madagascar [34, 35, 86, 125]. Sequencing and alignment of the atypical isolates from rat kidneys in Mayotte [86] showed perfect identity with isolates derived from people [35].

**Table 5:** *Leptospira* species^a^ reported in acute human leptospirosis and animal carrier hosts by African country.

	Human Studies	Animal Studies	
Species	Country	Host species	Country
***L*. *borgpetersenii***	Kenya[38, 39]	African grass rat (*Arvicanthus niloticus*)	Nigeria[87]
	Mayotte[34, 35]	Black rat (*Rattus rattus*)	Benin[103], Egypt[92], Mayotte[86]
		Cattle (*Bos* spp.)	Nigeria[87, 89–91], South Africa[121], Zimbabwe[98]
		Comoro rousette (*Rousettus oblivious*)	Comoros[107]
		Fringe-tailed gerbil (*Gerbilliscus robusta*)	Kenya[88]
		Giant African pouched rat (*Cricetomys gambianus*)	Tanzania[112]
		Lesser tufted-tailed rat (*Eliurus minor*)	Madagascar[125]
		Long-winged bats (*Miniopterus* spp)^b^	Madagascar[125]
		Madagascar free-tailed bat *(Otomops madagascariensis)*	Madagascar[107]
		Multimammate mouse (*Mastomys* sp.)	Benin[103]
		Pigs (*Sus scrofa domesticus*)	Tunisia[78]
		Shrew tenrecs (*Microgale* spp.)^c^	Madagascar[125]
		South African pouched mouse (*Saccostomys campestris*)	Kenya[88]
***L*. *borgpetersenii*-like** ^d^	Mayotte[35]	Black rat (*Rattus rattus*)	Mayotte[86]
		Shrew tenrec (*Microgale cowani*, *Microgale dobsoni*)	Madagascar[125]
***L*. *interrogans***	Egypt[56]	African giant shrew (*Crocidura oliveri*)	Benin[103]
	Ghana[44]	African grass rat (*Arvicanthus niloticus*)	Nigeria[87]
	Kenya[38, 39]	Asian house shrew (*Suncus murinus*)	Madagascar[118]
	Mayotte[34, 35]	Banded mongoose (*Mungo mungo*)	Botswana[105]
		Black rat (*Rattus rattus*)	Egypt[92], Mayotte[86], Madagascar[118]
		Brown rat (*Rattus norvegicus*)	Benin[103], Madagascar[118]
		Cattle (*Bos* spp.)	Botswana[102], Nigeria[87], South Africa[101], Zimbabwe[94]
		Comoro rousette (*Rousettus oblivious*)	Comoros[107]
		House mouse (*Mus musculus*)	Kenya[124], Madagascar[118]
		Pigs (*Sus scrofa domesticus*)	South Africa[104, 122]
		Rusty-bellied brush-furred rat (*Lophuromys sikapusi*)	Cameroon[109]
***L*. *kirschneri***	Egypt[56]	African grass rat (*Arvicanthus niloticus*)	Kenya[88]
	Kenya[39]	Black rat (*Rattus rattus*)	Mayotte[86]
	Mayotte[34, 35]	Cattle (*Bos* spp.)	Kenya[119], Tanzania[114], Zimbabwe[97]
		House mouse (*Mus musculus*)	Kenya[124]
		Shrew (*Crocidura* spp.)	Benin[103]
		Streaked tenrec (*Hemicentetes nigriceps*, *H*. *semispinosus*)	Madagascar[125]

Footnotes

^a^ methodology includes genetic typing of isolates, DNA sequencing following PCR detection, extrapolation of serovar data with species determined by reference to KIT *Leptospira* library.

^b^ *Miniopterus* spp. include *Miniopterus gleni*, *Miniopterus goudoti*, *Miniopterus griffithsi*, *Miniopterus mahafaliensis*, *Miniopterus majori*, *Miniopterus soroculus*

^c^ *Microgale* spp. include *Microgale longicaudata*, *Microgale majori*, *Microgale principula*

^d^ Described as *L*. *borgpetersenii*-like,[35] *L*. *borgpetersenii* Group B[86] and recently re-classified as *L*. *mayottensis*[134]

## Discussion

This systematic review is the first to synthesize and compile data on the epidemiology of acute human leptospirosis and pathogenic *Leptospira* spp. infection in animals in Africa. Leptospirosis remains amongst the neglected tropical diseases and is frequently overlooked in research priorities for African countries [1]. Yet, through this systematic review we have revealed a wealth of scientific evidence for acute human infection demonstrating that acute leptospirosis is an important cause of febrile illness in hospital patients across the African continent. Few studies providing population-level data on leptospirosis incidence in Africa were identified but available estimates indicate that the disease incidence is high in both island and mainland populations. In reports of human disease and animal infection, three predominant species, *Leptospira borgpetersenii*, *L*. *interrogans* and *L*. *kirschneri*, and a variety of *Leptospira* serogroups were diagnosed. *Leptospira* infection was reported in a wide range of domestic and wild animal species from across Africa but studies linking data on animal infections with studies of acute human disease were rare.

Acute leptospirosis was diagnosed in up to 19 8% of inpatients with non-specific febrile illness in hospital-based cohort studies conducted in several countries identified by this review. In sub-Saharan Africa, recent studies have highlighted that clinical over-diagnosis of malaria may conceal other aetiologies of febrile illness [20, 128]. Consistent with findings in other resource-limited tropical settings (e.g. South America [15, 129] and South-East Asia [130–132]), the evidence synthesised here demonstrates that acute leptospirosis infection is geographically widespread across the continent and should be considered as an important differential diagnosis for non-specific febrile illness in Africa.

Few estimates of leptospirosis incidence in Africa were identified by our review, revealing a key gap in research and surveillance outputs to date. The majority of incidence estimates identified came from the Indian Ocean islands where reports of annual incidence ranged from 3 1 to 101 cases per 100,000 people. In the African continent, the western Indian Ocean Islands appear to be the best-characterised region with regards to the human leptospirosis burden, possibly as a consequence of greater access to public health laboratories through French Territorial links [133]. We identified only one report of annual leptospirosis incidence from mainland Africa. This estimate of 75 to 102 cases per 100,000 people [74] was calculated from Tanzanian hospital-based surveillance data and is consistent with the WHO leptospirosis burden epidemiology reference group (LERG) predicted median African incidence of 95 5 cases per 100,000 [7]. At present, given the lack of population level data highlighted by LERG and by this review, estimates of the incidence of leptospirosis in Africa should be interpreted with care. However the data that are available from the continent indicate that the overall leptospirosis burden is likely to be high relative to other global regions. If the incidence figures identified by this review are close to the true burden of disease, up to 750,000 people in Africa will develop acute leptospirosis each year, representing a substantial disease burden that would far exceed current worldwide estimates (500,000 annual cases worldwide) [23].

Our review has revealed three predominant *Leptospira* species and a considerable diversity in reported pathogenic *Leptospira* serogroups in people and animals across the continent. Animal hosts, including livestock and invasive and indigenous rodent species, were reported for the majority of species and serogroups detected in human cases. However, there was poor geographical overlap in serogroup reporting between human and animal studies. Based on the findings of this review, we suggest that the major animal hosts of human-infecting serovars may vary across Africa and that both livestock and rodents may play important roles in human disease transmission. Few data were identified that described *Leptospira* spp. diversity in human cases and animal populations from the same country, and few studies attempted to link data on acute human leptospirosis with evidence of *Leptospira* infection in local animal populations. Studies on the Indian Ocean Islands of Mayotte and Madagascar were the exception to this. Isolates with unusual patterns of genetic and serological diversity, recently reclassified as a new pathogenic species *Leptospira mayottensis* [134], were detected from both human and black rat infections, implicating the black rat as the source of these human infections [35, 86]. These studies demonstrate the value of integrated human and animal research to identify sources and transmission routes of human leptospirosis, which can in turn help prioritise investment in disease prevention and control efforts.

The data included in this review most likely represents only the tip of the leptospirosis iceberg in Africa. Underreporting of leptospirosis is thought to be substantial and an overall lack of awareness about the disease and poor accessibility of diagnostic facilities are likely to contribute to this underreporting in Africa populations [135–137]. Patterns in reporting characteristics such as over-representation of study areas with greater research infrastructure, logistical connections or prior knowledge of a disease burden may also have resulted in reporting bias, particularly in assessing the geographic distribution of reports. We observed patient selection bias in some human studies, which limited the usefulness of reported prevalence data from these sources. Methodological limitations identified in this review include the use of the broad geographical search term ‘Africa’ rather than individual country names in our initial database searches. This approach may have missed eligible citations that are not indexed to the term ‘Africa’ in our selected databases. Our inclusion criteria may have created a bias towards more recent citations because of diagnostic technological advancements since the early era of our search period. Marked heterogeneity in methods and reporting criteria for serological diagnostic data prevented the meaningful synthesis and analysis of data on the reactive serogroups in human studies. We chose to include non-English language articles to allow inclusion of articles published in the colonial era, or in local language journals. Wherever possible, a proficient language speaker, in partnership with a study author, performed article translation. However, it is possible that some eligible studies may have been overlooked due to translation limitations.

Addressing the neglect of leptospirosis in Africa will be a major challenging for the future of leptospirosis research. Systematic review studies such as this can help to raise awareness of the human health threat of leptospirosis in Africa among researchers and policy makers. For medical clinicians, the non-specific presenting signs of acute leptospirosis in patients poses a substantial diagnostic challenge in developing countries where laboratory capacity rarely exists to diagnose the infection [18–20]. Hence, increasing clinician awareness and the development of treatment guidelines for the management of febrile patients should be a priority in resource-limited settings [138]. Integration of risk factor analysis in human cohort studies of febrile disease is also strongly advocated and would be a valuable next step in identifying groups at high risk of infection, and defining important animal to human transmission routes.

Knowledge of reservoir or carrier animal hosts is considered essential to understanding the epidemiology, transmission and control of leptospirosis in each setting [9, 11], yet our review has revealed that the linkages between *Leptospira* infections in people and animals are rarely addressed in the existing literature. Human and animal *Leptospira* infections are inextricably linked, and the multi-host epidemiology of leptospirosis means that there may be many potential sources of infection in a given setting. In the future, greater emphasis should be placed on performing multidisciplinary human and animal leptospirosis studies in the same geographical settings. Connecting investigations of animal reservoir populations with confirmed human cases would improve our understanding of the role that different animal species play in the transmission of pathogenic *Leptospira* serovars in a variety of geographic and environmental settings [8] [139]. Using an integrated ‘One Health’ approach to explore the relationship between human and animal *Leptospira* infection in areas where human disease is identified would also provide invaluable evidence to quantify the direct and indirect impacts of leptospirosis on human and animal populations in Africa [140, 141].

Control measures to prevent human leptospirosis often focus on rodent hosts of the disease. However, this review reveals that livestock are also important hosts of *Leptospira* infection in Africa, and may play a more substantial role in human disease transmission than is widely recognised. The clinical and sub-clinical productivity impacts of *Leptospira* infection in domestic animal populations in Africa are poorly understood. Around the world, several *Leptospira* serovars are considered to be of economic importance and cause production losses in a variety of livestock farming species including cattle, sheep, goats and pigs [17, 100, 142, 143]. More than 300 million of the world’s poorest people live in Africa, and at least 60% of these are in some part dependent on livestock for their livelihood [144]. Therefore, we consider that evaluating the impact of *Leptospira* infection on livestock health and productivity is also an important priority for prospective research in Africa. In the future, control of *Leptospira* infection in livestock species may have considerable scope to directly and indirectly improve human health and well-being in Africa, through reduced human leptospirosis transmission and increased productivity in livestock that subsistence farming communities [8, 142, 143, 145].

Finally, in 1967, the German leptospirosis researcher Kathe commented that ‘The world map of leptospirosis is, in fact, the world map of leptospirologists’ [67]. This is particularly true with regards to Africa. With this systematic review, we have started to outline the map of African leptospirosis; it is now time to fill in the gaps.

## Supporting Information

S1 Checklist PRISMA checklist. (DOC)

S1 File Study protocol: Epidemiology of Leptospirosis in Africa. (DOCX)

S1 Table Summary of included animal studies reporting confirmed cases of animal *Leptospira* spp. infection in Africa, 1930–2014. (DOCX)
