# Correction: Epidemiology of Leptospirosis in Africa: A Systematic Review of a Neglected Zoonosis and a Paradigm for 'One Health' in Africa

**DOI:** 10.1371/journal.pntd.0004552

**Published:** 2016-03-11

**Authors:** The PLOS Neglected Tropical Diseases Staff

There are a number of places in which decimal points are missing from numbers. This error is especially apparent in Table 3. Please see the corrected Table 3 here.

**Table 3:** Summary of eligible cohort and surveillance studies reporting human acute leptospirosis in Africa, 1930–2014

Citation	Country; Study year(s)	Setting and study design	Inclusion and exclusion criteria	Diagnostic tests	Number enrolled	Total number of eligible cases* (%)	No. of eligible confirmed & probable cases*
Van Riel et al[69]	DRC 1952–1954	Hospital; retrospective cohort	Clinical suspicion of leptospirosis	Culture (blood) in Vervoort-Korthoff media; Agglutination-lysis (MAT)	45	27 (60.0%)	5 confirmed; 22 probable
Kolochine-Erber & Brygoo[49]	Madagascar 1954–1955	Undefined; prospective cohort	Clinical suspicion of leptospirosis	Agglutination-lysis (MAT)	40	1 (2.5%)	1 probable
Forrester et al[42]	Kenya 1961–1962	Hospital; prospective cohort	Febrile illness unexplained by malaria, dysentery or pneumonia.	MAT	67	6 (9.0%)	All probable
Payet et al[61]	Senegal 1964–1965	Hospital; prospective cohort	Clinical suspicion of leptospirosis; mostly defined by jaundice	Agglutination-lysis (MAT)	53	3 (5.7%)	2 confirmed; 1 probable
Silverie et al[67]	Madagascar 1966–1967	Undefined; prospective cohort	Clinical suspicion of leptospirosis	Agglutination-lysis (MAT)	65	7 (10.8%)	All probable
De Geus et al[37]	Kenya 1967	Hospital and health centre; prospective cohort	Febrile illness (temperature ≥ 38°C) without obvious cause; negative malaria smear or no response to anti-malarial treatment	Culture (blood) in Fletcher’s and Cox’s media; MAT	39	7 (17.9%)	6 confirmed; 1 probable
Sankale et al[66]	Senegal 1967–1972	Hospital; retrospective cohort	Inpatients with serum samples tested for leptospirosis	Serum agglutination (MAT)	134	3 (2.2%)	All confirmed
De Geus et al[39]	Kenya 1968–1969	Hospital outpatient department and health centre; prospective cohort	Febrile illness (temperature ≥ 38.3°C) without obvious cause; negative malaria smear or no response to anti-malarial treatment ^a^	Culture (blood) in Fletcher’s media; MAT	91	10 (11.0%)	All confirmed
De Geus et al[38]	Kenya 1969	Hospital & outpatient department; prospective cohort & case-finding survey ^b^	Febrile illness (temperature ≥ 38.3°C) without obvious cause; negative malaria smear or no response to anti-malarial treatment	Culture (blood) in Fletcher’s media; MAT ^c^	281	9 (3.2%)	All confirmed
Kinebuchi et al[46]	Ghana NA	Hospital; prospective cohort	Clinical suspicion of leptospirosis, mostly defined by hepatitis or jaundice	Culture (blood) in Korthof’s media; MAT	99	13 (13.1%)	7 confirmed; 6 probable
Hogerzeil et al[44]	Ghana 1981–1982	Hospital outpatient department; prospective cohort	Group 1: fever without obvious cause and/or any of the following; jaundice, muscle pains, meningism, conjunctival injection, albuminuria; negative malaria smearGroup 2: jaundice	Culture (blood and urine) in Fletcher’s or EMJH media; MAT;IgM and IgG ELISA	Group 1: 88; Group 2: 102	Group 1: 4 (4.5%); Group 2: 2 (2.0%)	Group 1: 3 confirmed; 1 probable; Group 2: All confirmed
Delacollette et al[40]	DRC 1985–1986	Hospital; prospective cohort	Inpatients with black or red urine with confirmed haemoglobinuria	ELISA (unspecified)	38	1 (2.6%)	All probable
Pinn[63]	Seychelles 1988–1990	Hospital; prospective cohort	Inpatients with clinical diagnosis of leptospirosis ^d^	IgM ELISA	80	58 (72.5%)	All probable
Collares-Pereira et al[36]	Mozambique 1993	Hospital outpatient department; prospective cohort	Outpatients aged 18–50 years with acute febrile illness without obvious cause; negative malaria smear.	MAT	43	1 (2.3%)	1 probable
Yersin et al[70]	Seychelles 1995–1996	Nationwide health care providers; Prospective population-based surveillance	Fever or any of the following without obvious cause: myalgia, liver tenderness, jaundice, acute renal failure, bleeding tendency, radiographic lung infiltrates, or meningism	MAT; PCR (*rrs*)	125	75 (60.0%)	All confirmed
Desvars et al[41]	Reunion Island 1998–2008	Hospital; retrospective population-based surveillance	Cases voluntarily reported to Centre National de References de Leptospiroses (Paris, France)	Culture (blood), media not specified; MAT; PCR (target not specified)	NA	613 cases	All probable**
Ismail et al[45]	Egypt 1999–2003	Hospital; retrospective cohort	Group 1: fever (temperature ≥38°C) for ≥3 days in the absence of diarrhoea, pneumonia, typhoid fever, brucellosis or established fever of unknown origin.Group 2: acute hepatitis defined as signs of acute jaundice.	IgM ELISA (Pan-Bio); MAT	Group 1:886 ^e^; Group 2: 392 ^f^	Group 1: 141 (15.9%); Group 2: 63 (16.1%)	All probable**
Renault et al[64]	Réunion, 2004–2008	Hospital; retrospective population-based surveillance	Hospitalised cases of leptospirosis cases in Réunion reported to the Regional Directorate for Health and Social Affairs/Regional Health Agency of the Indian Ocean.	Confirmed cases: Culture (not specified), MAT or PCR (target not specified)Possible cases: IgM ELISA; MAT titre ≥ 1:50	240	160 (66.7%)	All probable**
Pages et al[73]	Réunion 2004–2012	Population-based surveillance	Confirmed or probable cases of leptospirosis in Réunion residents reported to the health watch platform of the French Regional Health Agency for the Indian Ocean.	Confirmed cases: Culture (not specified), MAT or PCR (target not specified)Possible cases: IgM ELISA.	NA	405 (Mean annual incidence = 5.6 per 100,000 population)	All probable**
Ari et al[31]	Kenya 2005	Community; prospective case-finding^g^	Community members with new onset febrile illness (temperature not defined) or joint pains	IgM ELISA (Pan-Bio)	12	3 (25.0%)	All probable
Bertherat et al[72]	DRC 2005	Community; retrospective case finding	Acute & convalescent patients with respiratory disease in a mining camp	MAT	82	8 (9.8%)	All probable
Parker et al[58]	Egypt 2005–2006	Hospital; prospective cohort	Fever ≥ 2 days or admission temperature ≥38.5°C; aged ≥ 4 years without obvious cause of fever, such as diarrhoea, pneumonia, or clinical diagnosis of typhoid fever or brucellosis.	Culture (blood) in EMJH; MAT; PCR; IgM ELISA	981	194 (19.8%)	45 confirmed; 149 probable
Parker et al[59]	Egypt 2005–2006	Hospital; prospective cohort	Fever ≥ 2 days or admission temperature ≥38.5°C; aged ≥ 4 years without obvious cause of fever; with laboratory evidence of co-infection with *Leptospira*, *Rickettsia typhi*, *Brucella*, or *Salmonellaenterica* serogroup Typhi	Culture (blood) in EMJH;MAT; PCR (*lig*A)	187 ^h^	152 (81.3%)	All confirmed
Murray et al[55, 56]	Egypt 2005–2007	Hospital; prospective cohort	Fever; aged ≥ 4 years without obvious cause of fever, such as diarrhoea, pneumonia, or clinical diagnosis of typhoid fever or brucellosis.	Culture (blood) in EMJH media; MAT; PCR (*lig*A)	2,441	98 (4.0%)	All probable**
Tagoe et al[68]	Ghana NA	Hospital; prospective cohort	Fever ≥ 2 days and temperature ≥38.0°C; aged ≥ 4 years without obvious cause of fever	IgM ELISA (Pan-Bio);MAT	166	13 (7.8%)	All probable
Biggs et al[33, 74]	Tanzania 2007–2008	Hospital; prospective cohort	Inpatients aged ≥13 years with fever (≥38.0°C oral) or inpatients aged 2 months to 12 years with history of fever within 48 hours or admission temperature ≥37.5°C axillary ≥38.0°C rectal.	MAT	831 total; 453 paired samples; 378 single samples	70 (8.4%);	40 confirmed; 30 probable
Bourhy et al[35]	Mayotte 2007–2008	Undefined; prospective cohort	Fever (temperature ≥38°C) for ≤7days and headache and/or myalgia	Culture (blood) in EMJH media; PCR (*rrs*)	388	53 (13.7%),	All confirmed
Bourhy et al[34]	Mayotte ^k^ 2007–2010	Undefined; population-based surveillance	Patients for which a blood sample was submitted for leptospirosis diagnosis to the Hospital Centre of Mayotte	Culture (blood) in EMJH media; PCR (*lbf1*, *lipL32*, *rrs*)	2,523	198 (7.8%)	All confirmed

*Figures reported here are based on the number of reported acute leptospirosis cases that met our review case definitions (see Table 1 for case definitions) and therefore may vary from the values reported in the original citations.

** All cases met probable case definitions. An unspecified proportion of positive cases also met the case definition for confirmed cases but exact numbers could not be determined from the available data.

a Patients who refused hospital admission were not investigated.

b Methods describe a change to a case-finding survey partway through the study, but full details not available

c MAT performed in a subset of participants only

d Clinical diagnosis defined as 3 of the following: headache or fever (temperature not defined), evidence of liver inflammation (defined as jaundice, tender liver, and/or abnormal liver function tests), evidence of renal inflammation (haematuria and/or abnormal renal function), or evidence of muscle inflammation (tenderness and/or elevated creatine phosphokinase)

e All tested negative for Salmonella enterica serovar Typhi, Brucella spp., and Rickettsia spp.

f All tested negative for Hepatitis A, B, and C.

g In setting of outbreak of acute febrile illness in a well-defined population

h 187 patients were diagnosed with selected co-infections out of a total cohort of 1510 patients with non-specific febrile illness.

ϖ Taken 9 days of onset of illness

k Also report two imported cases from Comoros and Madagascar respectively

Decimal points are also missing in several places in the text of the article. A space appears where a decimal point should be.
