# Supplementary material for: Epidemiology of Leptospirosis in Africa: A Systematic Review of a Neglected Zoonosis and a Paradigm for ‘One Health’ in Africa
Source: PLoS Negl Trop Dis. 2015 Sep 14;9(9):e0003899. doi: 10.1371/journal.pntd.0003899 (PMC4569256; doi:10.1371/journal.pntd.0003899)
Supplement: S1 File — (DOCX) [file pntd.0003899.s002.docx]

**Fig S1: Epidemiology of Leptospirosis in Africa: A Systematic Review of a Neglected Zoonosis and a Paradigm for ‘One Health’ in Africa**

**Study protocol**

**Search strategy:** Initial database searches were performed of PubMed, Web of Science, Biosis, CABI abstracts, Zoological Record, Africa-Wide NiPAD, and Africa Index Medicus for resources published between 1^st^ January 1930 – 31^st^ May 2013, and Embase (Ovid) for resourced published between 1947 -2013. An update search was performed on 24^th^ November 2014 to identify additional articles published in print or online between 1^st^ June 2013 and 31^st^ October 2014. Search terms were developed with guidance from a library scientist (Megan Von Isenberg). PubMed search terms included: (leptospirosis[mesh] OR leptospirosis[Text Word] OR leptospira[Text Word] OR leptospira[mesh) AND (africa[mesh] OR africa*[Text Word]). Embase search included: ((leptospirosis [sh] OR leptospira [sh] OR leptospirosis [tw] OR leptospira [tw]) AND (africa*[sh] OR africa*[tw])). For Web of Science, Biosis, CABI abstracts, and Zoological Record, we queried: Search box 1: Topic=(leptospirosis OR leptospira) AND search box 2: topic=(Africa*). Search terms for Africa-Wide NiPAD were (SU (leptospirosis OR leptospira) OR TX (leptospirosis OR leptospira)) AND (AB Africa* OR GE africa OR SU africa OR TI Africa* OR KW africa), and for Africa Index Medicus we searched using leptospirosis OR *Leptospira*. Two researchers (KJA, HMB) together de-duplicated the results in Microsoft Excel (Microsoft Corporation, Redmond, WA, USA) and determined the number of unique references from each database. Searches were updated on 24^th^ November 2014 using the same strategies to include articles published between June 2013 and October 2014. Additional articles for inclusion were identified by bibliography hand searches of relevant articles [1].

**Study selection and criteria: Abstract review**

Abstracts and titles were compiled in EndNote (Thomson Reuters, Philadelphia, PA, USA). The title and abstract for each unique citation was independently reviewed in a blinded standardized manner by two researchers (KJA, HMB) to determine whether it fit pre-determined inclusion and exclusion criteria. The UN macro-geographical definition of Africa was used to define the geographic boundaries of this review [2]. Abstracts were included if they presented data from any country within the UN definition of Africa and were published in any language historically associated with Africa, including English, French, Portuguese, Spanish, German, Dutch, Italian, or Afrikaans. We excluded any abstract that did not include original human or animal leptospirosis research data, including reviews, textbooks, letters to the editor, policy papers, and lay press and media stories. Additionally, we excluded any abstract in which did not investigate for the presence of naturally occurring cases of leptospirosis in human or animal populations including studies which described in vitro or in vivo experiments, laboratory methods descriptions or abstracts that described only environmental data. We excluded case reports of returned travellers given potential uncertainty around the specific location where infection was acquired. Citations for which no abstract was available moved onto the next stage of full text review. A third researcher (JEH) served as the tiebreaker for any discordant inclusion or exclusion decisions (between KJA and HMB) during the title and abstract review phase. All references deemed to meet title and abstract review criteria were carried forward for full text review. A full list of abstract exclusion criteria (with exclusion coding hierarchy used to generate Figure 1) is given below:

Title and abstract review exclusion criteria (with coding hierarchy)

1. Article type
   1. *Review or summary article without original data*
   2. *Editorial, letter to the editor opinion, commentary or policy article without original data*
   3. *Textbook or handbook rather than publication of new data*
   4. *Lay media publications or broadcasts*
   5. *Abstracts with corresponding full manuscripts with the same data*
2. Language: *exclude if title/abstract are NOT ENGLISH, FRENCH, PORTUGESE, SPANISH, DUTCH, GERMAN, ITALIAN, AFRIKAANS*
3. Geographic focus: *Exclude countries out-with the UN definition of Africa*
4. Topic focus
   1. *Wrong agent*
   2. *Experimental data (in vitro or in vivo cellular, molecular, biochemical or other studies that do not include naturally occurring cases of leptospirosis in humans or animals)*
   3. *Laboratory methods descriptions*
   4. *Leptospirosis included in the diagnostic evaluation or as a differential diagnosis, but diagnosis of leptospirosis infection or exposure was not reached*
   5. *Social science or environmental or climate modeling data only [no testing in either people or animals in the natural setting]*
   6. *Case reports of returned travelers (because origin of infection may be in doubt) [include all migration movements etc.]*

**Study selection and criteria: Full text review**

Articles eligible for inclusion following abstract review were retrieved in full text format. Definitions of acute human leptospirosis and animal infection were pre-defined and agreed by three authors (KJA, HMB, JEBH) in alignment with recommendations from WHO and international leptospirosis reference laboratories (see Table 1). Foreign language articles identified for full text review (n=97) included 83 French language articles translated by KJA with assistance from a native language speaker; 7 German language articles translated by a native language speaker; 4 Italian articles translated by a native language speaker; 2 Afrikaans and 1 Dutch language articles, which were translated using online translation software with support from a Dutch language speaker [3].(Google Translate, Mountain View, California, USA). Full text articles were assessed against detailed full text criteria given below:

**Full-text exclusion criteria (with coding hierarchy)**

1. Meets any of the abstract review exclusion criteria
   1. *Any texts where the country is NOT specified should also be excluded under abstract exclusion criteria 3*
2. Full text article does NOT meet criteria/case definitions (Table 2) for either of the study target populations (see accompanying definitions):
   1. *Acutely ill people*
   2. *Carrier animals*
3. Reports on the same cohort report in other, more comprehensive studies
   1. *Hierarchy of these documents:*
   2. *Peer-reviewed publication > grey literature: e.g. Data comprehensively published in peer-review journal would be accepted in preference to a conference abstract of the data or internal report of the same data published at an earlier date.*
   3. *Comprehensive paper > partial report: e.g. if two peer-reviewed papers are published which contain overlapping data, the most comprehensive text should be selected for inclusion.*
   4. *Finally; if two identical papers are published in different sources, the earliest paper/first report – based on the date of publication – should be selected for inclusion.*
4. Methods or study population not described in sufficient detail to determine whether the study meets inclusion or exclusion criteria

**Data extraction and synthesis:** Two investigators (KJA, HMB) individually extracted pre-determined qualitative and quantitative data from each eligible citation and entered into two (human and animal) Microsoft Excel spread sheets. Extracted data included:

1. Geographical locations (country, locality);
2. Study year and duration;
3. Setting, population and study type
   1. *Human*:
      1. Prospective or retrospective cohort study
      2. Population-based surveillance,
      3. Community surveillance
      4. Case report or series;
   2. *Animal*:
      1. Abattoir surveillance
      2. Domestic animal surveillance
      3. Wild animal surveillance
      4. Disease outbreaks
4. Study inclusion criteria; e.g. febrile disease, icteric disease, clinical suspicion of leptospirosis
5. Study diagnostic methodology and case definition; by comparison to predefined case definition criteria given in Table 2.
6. Numbers and species of tested (animals only);
7. Number of positive cases
   1. *Human*: confirmed vs. probable;
   2. *Animal*: confirmed;
8. Prevalence
   1. *Human*: From prospective hospital cohort studies or community-based surveillance; excluding studies that cited clinical suspicion of leptospirosis as a patient selection criteria
   2. *Animal*: surveillance studies
9. Incidence (human, population-based studies only);
10. Diversity of serological diagnostic panels (human only);
11. Predominant reactive serogroups (human only);
12. Serological and genetic isolate typing (derived from human patients or carrier animals).

**Critical assessment of methodological quality and bias:** The risk of bias in included studies such as selection or reporting bias was assessed following an adaptation of the Cochrane guidelines for systematic reviews of medical interventions [4]. Full text study validity and methodological quality was assessed by comparison to pre-determined case definition criteria to control for heterogeneity in study design and diagnostic methodology (Table 2). Other points for assessment of methodological quality and bias for individual studies was performed considering the following points:

1. Selection bias:
   1. Study design e.g. targeted outbreak sampling vs. prospective cohort study (human) or cross-sectional surveillance (animal)
   2. Patient selection strategy including study cohort selection criteria
2. Detection bias:
   1. Evaluation and comparison of diagnostic tests used within the study and between cohorts of patients.
   2. Assessment of case definitions used by individual studies
3. Attrition bias:
   1. Assessment of completeness of reporting of study data and diagnostic results

Studies classified as high-risk for bias were not included in quantitative analysis of leptospirosis prevalence and incidence data. In studies with incomplete reporting of case definitions and diagnostic criteria, we only included data from patients or cohorts where we considered that a valid assessment of the methodological quality could be performed. For citations where only a subset of reported positives met our study criteria, prevalence was re-calculated after data adjustment.

References

1. Hopewell S, Clarke M, Lefebvre C, Scherer R. Handsearching versus electronic searching to identify reports of randomized trials. The Cochrane database of systematic reviews. 2007;(2):Mr000001. Epub 2007/04/20. doi: 10.1002/14651858.MR000001.pub2. PubMed PMID: 17443625.

2. United Nations Statistics Division. Composition of macro geographical (continental) regions, geographical sub-regions, and selected economic and other groupings [Website]. 2012 [cited 2014 3rd February]. Available from: <http://unstats.un.org/unsd/methods/m49/m49regin.htm>.

3. Google Translate. <http://www.translate.google.co.uk> Mountain View, California, USA [cited 2013 3rd June ]. Available from: <http://translate.google.com>.

4. Higgins JP, Green S. Cochrane handbook for systematic reviews of interventions: Wiley Online Library; 2008.
