# Supplementary material for: Epidemiology of Leptospirosis in Africa: A Systematic Review of a Neglected Zoonosis and a Paradigm for ‘One Health’ in Africa
Source: PLoS Negl Trop Dis. 2015 Sep 14;9(9):e0003899. doi: 10.1371/journal.pntd.0003899 (PMC4569256; doi:10.1371/journal.pntd.0003899)
Supplement: S1 Table — (DOCX) [file pntd.0003899.s003.docx]

**Supplementary material:** **Epidemiology of Leptospirosis in Africa: A systematic review of a neglected zoonosis and a paradigm for “One Health” in Africa**

S1 Table: Summary of included animal studies reporting confirmed cases of animal *Leptospira* spp. infection in Africa, 1930 – 2014

| **Citation** | **Country; Study year(s)** | **Study design** | **Diagnostic tests (See Case definitions in Table 2)** | **Animal Species tested** | **Number tested** | **Total number of infected individuals (Prevalence %)** |
| --- | --- | --- | --- | --- | --- | --- |
| Brownlow[1] | Egypt 1959 | Wild animal surveillance | Culture (Kidney); Stuart’s media | House mouse (*Mus musculus*) | 44 | 2 (4·5%) |
| Lazuga & Bonnefous[2] | Tunisia NA | Wild animal surveillance | Culture (Kidney); Korthoff’s media | Brown rat (*Rattus norvegicus*) | 919 | 57 (6·2%) |
| Ball[3] | Kenya 1963 | Wild animal surveillance | Culture (Kidney); Cox’s media | Fringe-tailed gerbil (*Gerbilliscus robustus*) | 113 | 4 (3·5%) |
| Rademan et al.[4] | South Africa 1963-1964 | Wild animal surveillance | Culture (Kidney); Korthoff’s media | Brown rat (*Rattus norvegicus*) | 256 | 7 (2·7%) |
| Chadli & Bakoss[5] | Tunisia 1964 | Wild animal surveillance | Culture (Kidney); Korthoff’s media | Brown rat (*Rattus norvegicus*) | 130 | 11 (8·5%) |
| Bakoss; Bakoss & Chadli[6, 7] | Tunisia 1965 | Abattoir surveillance and strain typing | Culture (Kidney); Korthoff’s media | Pigs (*Sus scrofa domesticus*) | 185 | 2 (1·1%) |
|  |  |  |  | Fringe-tailed gerbil (*Gerbilliscus robustus)* | NA | 16 isolates obtained; breakdown not given |
| Dikken et al.[8] | Kenya 1967-1968 | Strain typing of strains from naturally-occurring infection | Culture (Kidney); media not specified. | African grass rat (*Arvicanthus niloticus*) | NA |  |
|  |  |  |  | South African pouched mouse (*Saccostomys campestris*) | NA |  |
| Maronpot et al.[9] | Egypt NA | Domestic animal surveillance | Culture (Urine); Fletcher’s & Ellinghausen’s media | Dogs (*Canis lupis familiaris*) | 68 | 2 (2·9%) |
|  |  |  |  | House mouse (*Mus musculus*) | 95 | 7 (7·6%) |
| Barsoum[10] | Egypt NA | Wild animal surveillance | Culture (Kidney; kidney &u urine pooled (*M. musculus* only)); Ellinghausen liquid media; Fletcher’s media | Egyptian Mongoose (*Herpestes ichneumon*) | 16 | 2 (12·5%) |
|  |  |  |  | Red fox (*Vulpes vulpes*) | 16 | 1 (6·3%) |
| Van Rensburg[11] | South Africa NA | Animal disease outbreak | Culture (Kidney); Korthoff’s media | Pigs (*Sus scrofa domesticus*) | 10 | 6 (60·0%) |
| Diallo & Dennis[12] | Nigeria 1974-1976 | Wild animal surveillance & abattoir sampling | Culture (Kidney); Ellinghausen-McCullough media | African grass rat (*Arvicanthus niloticus*) | 221 | 8 (3·6%) |
|  |  |  |  | Cattle (*Bos* sp.) | 74 | 5 (6·8%) |
| Tabel & Losos[13] | Kenya 1975-1976 | Animal disease outbreak | Culture (Kidney); Korthoff’s media | Cattle (*Bos* sp.) | 9 | 2 (22·2%) |
| Le Bras et al.[14] | Cameroon 1975-1976 | Wild animal surveillance | Culture (Kidney); Korthoff’s media | Rusty-bellied brush-furred rat *Lophuromys sikapusi*) | NA | 1 (NA) |
| Herr et al.[15] | South Africa 1980 | Animal disease outbreak | Culture (Urine); Semi-solid Stuart’s & EMJH media | Cattle (*Bos* sp.) | 20 | 10 (50·0%) |
| Mugarula[16] | Tanzania 1980 | Domestic animal surveillance | Culture (Urine); Korthoff’s media | Dogs (*Canis lupis familiaris*) | 3693 | 48 (1·3%) |
| Herr & Winnen[17] | Botswana NA | Animal disease outbreak | Culture (Urine); EMJH media | Cattle (*Bos* sp.) | 40 | 1 (2·5%) |
| Ezeh et al.[18-20] | Nigeria 1984-1985 | Abattoir sampling & strain typing | Culture (Kidney); EMJH media | Cattle (*Bos* sp.) | 525 | 6 (1·1%) |
| Te Brugge & Dreyer[21] | South Africa NA | Animal disease outbreak | Culture (Urine); EMJH media | Cattle (*Bos* sp.) | 19 | 3 (15·8%) |
| de Lange et al.[22] | South Africa NA | Animal disease outbreak investigation | Culture (Kidney, renal lymph node, tissue from aborted foetuses); EMJH media | Pigs (*Sus scrofa domesticus*) | 14 | 13 (92·9%) |
| Hunter et al.[23] | South Africa NA | Abattoir surveillance | Culture (Kidney); EMJH media | Pigs (*Sus scrofa domesticus*) | 30 | 20 (66·6%) |
| Feresu et al.[24-30] | Zimbabwe 1987-1988 | Abattoir surveillance and strain typing | Culture (Kidney); EMJH media | Cattle (*Bos* sp.) | 480 | 50 (10·4%) |
|  |  |  |  | Black rat (*Rattus rattus*) | 293 | 46 positive samples § |
| Dalu & Feresu[31] | Zimbabwe 1995-1996 | Wild animal surveillance | Culture (Kidney, Urine); EMJH media | Multimammate mouse (*Mastomys natalensis*) | 85 | 2 positive samples § |
|  |  |  |  | House mouse (*Mus musculus*) | 3 | 4 positive samples § |
| Machang’u et al.[32] | Tanzania NA | Abattoir surveillance | Culture (Urine); Fletcher’s media | Cattle (*Bos* sp.) | 1021 | 7 (0·1%) |
| Gummow et al.[33] | South Africa NA | Animal disease outbreak | Culture (Kidney, aborted foetuses, bovine urine); medium not stated | Pigs (*Sus scrofa domesticus*) | 13 | 12 (92·3%) |
|  |  |  |  | Cattle (*Bos* sp.) | 12 | 3 (25·0%) |
| Machang’u et al.[34, 35] | Tanzania NA | Strain typing | Culture (Urine); Fletcher’s media | Giant African pouched rat (*Cricetomys gambianus*) | 83 | 8 (9.6%) |
|  |  |  |  | Brown rat (*Rattus norvegicus)* | 63 | 8 (12·7%) |
| Taylor et al.[36] | South Africa 2004-2005 | Wild animal surveillance | PCR (Kidney); 16S ribosomal genes (*rrs*)[37] | House mouse (*Mus musculus*) | 2 | 1 (50·0%) |
|  |  |  |  | Black rat (*Rattus rattus*) | 2 | 1 (50·0%) |
| Zimmermann et al.[38] | Guinea 2004 | Investigation of animals following a human disease outbreak | PCR (Kidney): Primer target not described | Rodents; various species^#^ | 330 | 5 (1.5%) |
| Mgode et al.[39] | Tanzania NA | Wild animal surveillance | PCR (Kidney); 16S ribosomal genes (*rrs*)[37] | Multimammate mice (*Mastomys* spp.) | 18 | PCR: 1 (6·3%) |
|  |  |  | Culture (Kidney); Fletcher’s media | Shrews (*Crocidura* spp.) | 7 | PCR: 2 (28·6%) |
|  |  |  |  |  |  | Culture: 2 (28.6%) |
| Mgode et al.[40] | Tanzania NA | Strain typing | Culture (Urine); Fletcher & EMJH media | Cattle (*Bos* sp.) | Not given | Not given |
| Felt et al.[41] | Egypt 2006-2007 | Wild animal surveillance | PCR (Kidney); LIPs60;[42] *lig A* & *lig B*[43] | Black rats (*Rattus rattus)* | 100 | PCR: 11 (11·0%)$ |
|  |  |  | Culture (Kidney, urine, blood); EMJH media. |  |  | Culture: 4 (4·0%) |
| Desvars et al.[44] | Mayotte 2007 | Animal surveillance resulting from human disease outbreak | qPCR (Kidney); *lipL32*[45] | Black rats (*Rattus rattus*) | 141 | 42 (29·8%) |
| Kessy et al.[46] | Tanzania 2007-2008 | Abattoir surveillance | Culture (Kidney & urine); Fletcher’s media | Pigs (*Sus scrofa domesticus*) | 236 | 2 (0·8%) |
|  |  |  |  | House mouse (*Mus musculus*) | 194 | 37 (19·1%) |
| Halliday et al.[47] | Kenya 2008 | Wild animal surveillance | qPCR (Kidney); *secY*[48] | Brown rat (*Rattus norvegicus*) | 10 | 1 (10·0%) |
|  |  |  |  | Black rat (*Rattus rattus*) | 33 | 3 (9·1%) |
|  |  |  |  | House mouse *(Mus musculus)* | 55 | PCR: 5 (10.0%)* |
|  |  |  |  |  |  | Culture: 0 (0%)* |
| Rahelinirina et al.[49] | Madagascar 2008-2009 | Wild animal surveillance | Culture (Kidney* & urine); EMJH media | Brown rat (*Rattus norvegicus*) | 96 | PCR: 39 (40·6%)* |
|  |  |  |  |  |  | Culture: 6 (6·3%)* |
|  |  |  | qPCR (Kidney* & urine); *Hap1/lipL32*[50] | Black rat (*Rattus rattus*) | 94 | PCR: 27 (28.7%)* |
|  |  |  |  |  |  | Culture: 3 (3·2%)* |
|  |  |  |  | Asian house shrew (*Suncus murinus*) | 23 | PCR: 10 (43.5%)* |
|  |  |  |  |  |  | Culture: 0 (0%)* |
|  |  |  |  | House mouse (*Mus musculus*) | 13^ | 11 (84.6%) |
|  |  |  |  | Black rat (*Rattus rattus*) | 76^ | 50 (65.8%) |
|  |  |  |  | Brown rat (*Rattus norvegicus*) | 6^ | 4 (66.6%) |
|  |  |  |  | Asian House Shrew (*Suncus murinus*) | 48^ | 15 (31.2%) |
|  |  |  |  | Dog (*Canis lupis familiaris*) | 24^ | 7 (29.2%) |
| Desvars et al.[51] | Réunion 2009 | Wild animal surveillance and domestic animal surveillance | qPCR (Kidney; urine from bats only); *lipL32*[45] | Cat (*Felis cattus*) | 21^ | 6 (28.6%) |
|  |  |  |  | Cattle (*Bos* sp.) | 77^ | 14 (18.2%) |
|  |  |  |  | Goat (*Capra aegagrus hircus*) | 49^ | 13 (26.5%) |
|  |  |  |  | Rusa Deer (*Rusa timorensis*) | 32^ | 6 (18.8%) |
|  |  |  |  | Pigs*Sus scrofa domesticus*) | 83^ | 13 (15.6%) |
|  |  |  |  | Bats (*Mormopterus francoismoutoui*) | 2^ | 2 (100%) |
|  |  |  |  | Multimammate mice (*Mastomys* spp.) | 12 | 4 (33.3%) |
|  |  |  |  | Brown rats (*Rattus norvegicus*) | 11 | 3 (27.3%) |
| Houemenou et al.[52] | Benin 2009 | Wild animal surveillance | qPCR (Kidney); *secY* [48] | Black rats (*Rattus rattus*) | 60 | 8 (13.3%) |
|  |  |  |  | African giant shrew (*Crocidura olivieri*i) | 6 | 1 (16.7%) |
|  |  |  |  | Shrew (*Crocidura* spp.) | 1 | 1 (100%) |
| Jobbins et al.[53] | Botswana 2009-2012 | Wild animal surveillance | PCR (Kidney); 23S rDNA gene target (*rrl*) [54] | Banded mongoose (*Mungos mungo)* | 41 | 17 (41.4%) |
|  |  |  |  | Selous mongoose (*Paracynictis selousi*) | 1 | 1 (100·0%) |
| Lagadec et al.[55] | Madagascar, Comoros NA | Wild animal surveillance | qPCR (pooled kidney, spleen & lung); 16S rRNA (*rrs*)[56] | Bats; various species^&^ | 129 | 27 (20.9%) |
|  |  |  |  | Lesser tufted-tailed rat (*Eliurus minor*) | 112 | 32 (28·6%) |
|  |  |  |  | Cowan’s shrew tenrec (*Microgale cowani*) | 72 | 2 (2·8%) |
|  |  |  |  | Dobson’s shrew tenrec (*Microgale dobsoni*) | 54 | 3 (5·6%) |
|  |  |  |  | Lesser long-tailed shrew tenrec (*Microgale longicaudata*) | 12 | 1 (8·3%) |
| Dietrich et al.[57] | Madagascar (2010-2012) | Wild animal surveillance | qPCR (pooled kidney, spleen & lung); 16S rRNA (*rrs*)[56] | Major’s long-tailed tenrec (*Microgale majori*) | 10 | 2 (20·0%) |
|  |  |  |  | Greater long-tailed shrew tenrec (*Microgale principula*) | 6 | 2 (33·3%) |
|  |  |  |  | Lowland streaked tenrec (*Hemicentetes semispinosus*) | 4 | 1 (25·0%) |
|  |  |  |  | Highland streaked tenrec (*Hemicentetes nigriceps*) | 12 | 1 (8·3%) |
|  |  |  |  | Bats; *Miniopterus* species && | NA | 6 (NA) |
| Nimo Paintsil et al.[58] | Ghana NA | Wild animal surveillance | PCR (kidney); not specificd | *Crocidura sp*. | NA | 1 (NA) |
| Hatem et al.[59] | Egypt NA | Wild animal surveillance | Culture (Rats: kidney; cattle: blood, milk and/or urine); EMJH media | Rats (Species not stated) | 200 | 9 (4·5%) |
|  |  | Domestic animal surveillance |  | Cattle (*Bos* sp.) | 625 | 7 (1·1%) |

**Footnotes**:

$ Numbers adjusted to report results for pathogenic *Leptospira* spp. only

§ Prevalence of carriers cannot be calculated due to duplication of results (i.e.kidney and urine tested for same animals but not distinguished in reporting)

# Various rodent species include: *Rattus rattus, Mus musculus, Crocidura* spp., *Mastomys* spp.; breakdown not given

* Only kidney results reported here. See original reference for full breakdown of positives by sample type.

^ Samples with PCR inhibition are excluded from denominator data

&Various bat species include: *Chaerephon pusillus, Miniopterus gleni, Miniopterus griffithsi, Miniopterus griveaudi, Miniopterus mahafaliensis, Mormopterus francoismoutoui, Mormopterus jugularis, Mytotis goudoti,* *Otomops madagascariensis, Rousettus obliviosus, Triaenops furculus, Triaenops menamena*

&&*Miniopterus* species includes: *M. gleni,* *M. goudoti, M. griffithsi, M. mahafaliensis, M. majori, M. soroculus,.*

1. Brownlow WJ, Dedeaux JD. Leptospirosis in animals of upper [southern] Egypt. Am J Trop Med Hyg. 1964;13(2):311–18. PubMed PMID: BIOSIS:PREV19644500088003.

2. Lazuga K, Bonnefous S. Contribution to the study of leptospirosis in rats in the city of Tunis [English and German summ.]. Arch Inst Pasteur Tunis. 1962;39(1):49–63. PubMed PMID: BIOSIS:PREV19634200002640.

3. Ball MG. Animal hosts of leptospires in Kenya and Uganda. Am J Trop Med Hyg. 1966;15(4):523–30. Epub 1966/07/01. PubMed PMID: 4957422.

4. Rademan J, Steytler JG, Wright N. First isolations of Leptospirae in Cape Town. S Afr Med J. 1964;38(30):694–96. PubMed PMID: BIOSIS:PREV19654600030698.

5. Chadli A, Bakoss P. Enquete sur la leptospirose en Tunisie. Arch Inst Pasteur Tunis. 1965;42(1):45–58. PubMed PMID: BIOSIS:PREV19674800118105.

6. Bakoss P. Leptospira tunis, a new serotype of the tarassovi group. Arch Inst Pasteur Tunis. 1969;46:17–23. PubMed PMID: CABI:19702200038.

7. Bakoss P, Chadli A. The pig, reservoir of Leptospira mitis in Tunisia. Arch Inst Pasteur Tunis. 1965;42(1):85–91. PubMed PMID: BIOSIS:PREV19674800118103.

8. Dikken H, Timmer VE, Njenga R. Three new leptospiral serovars from Kenya. Trop Geogr Med. 1981;33(4):343–6. Epub 1981/12/01. PubMed PMID: 7342381.

9. Maronpot RR, Barsoum IS, Ezzat E. Canine leptospirosis in Cairo. J Infect Dis. 1971;123:548–50. PubMed PMID: CABI:19712205209.

10. Barsoum IS, Moch RW, Botros BA, Kaiser MN. Leptospires isolated from wild mammals in Egypt. Trop Geogr Med. 1973;25(4):362–64. Epub 1973/12/01. PubMed PMID: 4786650.

11. Van Rensburg WJJ. Isolation of Leptospira-Canicola in Pigs and Dogs in South Africa. J S Afr Vet Assoc. 1973;44(4):435–36. PubMed PMID: BIOSIS:PREV197559003060.

12. Diallo AA, Dennis SM. Bacteriological survey of leptospirosis in Zaria, Nigeria. Trop Geogr Med. 1982;34(1):29–34. Epub 1982/03/01. PubMed PMID: 7043824.

13. Tabel H, Losos G. Report on an outbreak of bovine leptospirosis in Kenya due to Leptospira grippotyphosa. Bull Anim Health Prod Afr. 1979;27(1):61–4. PubMed PMID: CABI:19792253592.

14. Le Bras J, Guyer B, Sulzer C, Mailloux M. [Anademic focus of leptospirosis at Fondem (U.R. of Cameroon)]. Bull Soc Pathol Exot Filiales. 1977;70(6):569–83. Epub 1977/11/01. PubMed PMID: 615682.

15. Herr S, Riley AE, Neser JA, Roux D, De Lange JD. Leptospira Interrogans Ssp Pomona Associated with Abortion in Cattle Isolation Methods and Laboratory Animal Histo Pathology. Onderstepoort J Vet Res. 1982;49(1):57–62. PubMed PMID: BIOSIS:PREV198375019194.

16. Mugarula DR. Canine leptospirosis in Tabora township [Tanzania]. Bull Anim Health Prod Afr. 1984;32(1):99–101. PubMed PMID: CABI:19852269350.

17. Herr S, Winnen GM. First isolation of Leptospira interrogans serovar pomona from cattle in Botswana. J S Afr Vet Assoc. 1983;54(2):83–4. PubMed PMID: CABI:19852255671.

18. Ezeh AO, Ellis WA, Kmety E, Adesiyun AA, Addo PB. Bacteriological examination of bovine kidneys for leptospires in Plateau State, Nigeria. Rev Sci Tech. 1989;8(4):1005–8. PubMed PMID: CABI:19902203617.

19. Ezeh AO, Kmety E, Ellis WA, Addo PB, Adesiyun AA. Characterisation of leptospires isolated from cattle and man in Plateau State, Nigeria. Rev Sci Tech. 1989;8(4):1009–20. PubMed PMID: CABI:19902203618.

20. Ezeh AO, Kmety E, Ellis WA, Addo PB, Adesiyun AA. A new leptospiral serovar in the Pyrogenes serogroup isolated in Nigeria. Rev Sci Tech. 1990;9(4):1195–96. Epub 1990/12/01. PubMed PMID: 2132712.

21. Te Brugge LA, Dreyer T. Leptospira-Interrogans Serovar Hardjo Associated with Bovine Abortion in South Africa. Onderstepoort J Vet Res. 1985;52(1):51–2. PubMed PMID: BIOSIS:PREV198681063504.

22. de Lange JF, Gummow B, Turner GV, Redman AR. The isolation of Leptospira interrogans serovar pomona and related serological findings associated with a mixed farming unit in the Transvaal. Onderstepoort J Vet Res. 1987;54(2):119–21. Epub 1987/06/01. PubMed PMID: 3627726.

23. Hunter P, van der Vyver FH, Selmer-Olsen A, Henton MM, Herr S, de Lange JF. Leptospirosis as a cause of "white spot" kidneys in South African pig abattoirs. Onderstepoort J Vet Res. 1987;54(1):59–62. Epub 1987/03/01. PubMed PMID: 3587928.

24. Feresu SB. Isolation of Leptospira interrogans from kidneys of Zimbabwe beef cattle. Vet Rec. 1992;130(20):446–48. Epub 1992/05/16. PubMed PMID: 1621343.

25. Feresu SB, Bolin CA, Korver H. A new leptospiral serovar in the Icterohaemorrhagiae serogroup isolated from an ox in Zimbabwe. Int J Syst Bacteriol. 1993;43(1):179–82. PubMed PMID: CABI:19932279409.

26. Feresu SB, Bolin CA, Korver H. A new leptospiral serovar, ngavi, in the Tarassovi serogroup isolated from Zimbabwe oxen. Int J Syst Bacteriol. 1998;48(1):207–13. Epub 1998/05/02. PubMed PMID: 9542090.

27. Feresu SB, Bolin CA, Korver H, Kemp Hvd. Identification of leptospires of the Pomona and Grippotyphosa serogroups isolated from cattle in Zimbabwe. Res Vet Sci. 1995;59(1):92–4. PubMed PMID: CABI:19962204485.

28. Feresu SB, Bolin CA, Korver H, Terpstra WJ. Classification of leptospires of the pyrogenes serogroup isolated from cattle in Zimbabwe by cross-agglutinin absorption and restriction fragment length polymorphism analysis. Int J Syst Bacteriol. 1994;44(3):541–46. Epub 1994/07/01. PubMed PMID: 7915129.

29. Feresu SB, Korver H, Riquelme N, Baranton G, Bolin CA. Two new leptospiral serovars in the Hebdomadis serogroup isolated from Zimbabwe cattle. Int J Syst Bacteriol. 1996;46(3):694–98. Epub 1996/07/01. PubMed PMID: 8782678.

30. Feresu SB, Bolin CA, van de Kemp H, Korver H. Identification of a serogroup bataviae Leptospira strain isolated from an ox in Zimbabwe. Zentralbl Bakteriol. 1999;289(1):19–29. Epub 1999/03/30. PubMed PMID: 10096163.

31. Dalu JM, Feresu SB. Domestic rodents as reservoirs of pathogenic Leptospira on two city of Harare farms: Preliminary results of bacteriological and serological studies. Belg J Zool. 1997;127(Suppl.):105–12. PubMed PMID: BIOSIS:PREV199800085264.

32. Machang'u RS, Mgode G, Mpanduji D. Leptospirosis in animals and humans in selected areas of Tanzania. Belg J Zool. 1997;127(Suppl.1):97–104. PubMed PMID: CABI:19982214238.

33. Gummow B, Myburgh JG, Thompson PN, van der Lugt JJ, Spencer BT. Three case studies involving Leptospira interrogans serovar pomona infection in mixed farming units. J S Afr Vet Assoc. 1999;70(1):29–34. Epub 2000/06/16. PubMed PMID: 10855820.

34. Machang'u R, Mgode G, Asenga J, Mhamphi G, Hartskeerl R, Goris M, et al. Characterisation of Leptospira isolates from captive giant African pouched rats, Cricetomys gambianus. ACIAR Monograph Series. 2002;96:40–2. PubMed PMID: ZOOREC:ZOOR13900051607.

35. Machang'u RS, Mgode GF, Assenga J, Mhamphi G, Weetjens B, Cox C, et al. Serological and molecular characterization of leptospira serovar Kenya from captive African giant pouched rats (Cricetomys gambianus) from Morogoro Tanzania. FEMS Immunol Med Microbiol. 2004;41(2):117–21. doi: 10.1016/j.femsim.2004.02.002.

36. Taylor PJ, Arntzen L, Hayter M, Iles M, Frean J, Belmain S. Understanding and managing sanitary risks due to rodent zoonoses in an African city: beyond the Boston Model. Integr Zool. 2008;3(1):38–50. Epub 2008/03/01. doi: 10.1111/j.1749-4877.2008.00072.x. PubMed PMID: 21396050.

37. Murgia R, Riquelme N, Baranton G, Cinco M. Oligonucleotides specific for pathogenic and saprophytic leptospira occurring in water. FEMS Microbiol Lett. 1997;148(1):27–34.

38. Zimmermann S, ter Meulen A, Calvet E, Koivogui L, Sylla O, Goris M, et al. Seroprevalence and reservoirs of leptospirosis in Conakry (Guinea). Int J Antimicrob Agents. 2007;29(Suppl. 2):S49. PubMed PMID: BIOSIS:PREV200800320879.

39. Mgode GF, Mhamphi G, Katakweba A, Paemelaere E, Willekens N, Leirs H, et al. PCR detection of Leptospira DNA in rodents and insectivores from Tanzania. Belg J Zool. 2005;135:17–9. PubMed PMID: ZOOREC:ZOOR14501003047.

40. Mgode GF, Machang'u RS, Goris MG, Engelbert M, Sondij S, Hartskeerl RA. New Leptospira serovar Sokoine of serogroup Icterohaemorrhagiae from cattle in Tanzania. Int J Syst Evol Microbiol. 2006;56(3):593–7. doi: 10.1099/ijs.0.63278-0.

41. Felt SA, Wasfy MO, El-Tras WF, Samir A, Rahaman BA, Boshra M, et al. Cross-species surveillance of Leptospira in domestic and peri-domestic animals in Mahalla City, Gharbeya Governorate, Egypt. Am J Trop Med Hyg. 2011;84(3):420–25. Epub 2011/03/03. doi: 10.4269/ajtmh.2011.10-0393. PubMed PMID: 21363980; PubMed Central PMCID: PMC3042818.

42. Gravekamp C, Van de Kemp H, Franzen M, Carrington D, Schoone G, Van Eys G, et al. Detection of seven species of pathogenic leptospires by PCR using two sets of primers. J Gen Microbiol. 1993;139(8):1691–700.

43. Palaniappan RU, Chang Y-F, Chang C-F, Pan M, Yang C, Harpending P, et al. Evaluation of *lig*-based conventional and real time PCR for the detection of pathogenic leptospires. Mol Cell Probes. 2005;19(2):111–17.

44. Desvars A, Naze F, Vourc'h G, Cardinale E, Picardeau M, Michault A, et al. Similarities in Leptospira serogroup and species distribution in animals and humans in the Indian Ocean island of Mayotte. Am J Trop Med Hyg. 2012;87(1):134–40. PubMed PMID: CABI:20123277776.

45. Stoddard RA, Gee JE, Wilkins PP, McCaustland K, Hoffmaster AR. Detection of pathogenic *Leptospira* spp. through TaqMan polymerase chain reaction targeting the LipL32 gene. Diagn Microbiol Infect Dis. 2009;64(3):247–55.

46. Kessy MJ, Machang'u RS, Swai ES. A microbiological and serological study of leptospirosis among pigs in the Morogoro municipality, Tanzania. Trop Anim Health Prod. 2010;42(3):523–30. Epub 2009/09/19. doi: 10.1007/s11250-009-9455-z. PubMed PMID: 19763865.

47. Halliday JE, Knobel DL, Allan KJ, de CBBM, Handel I, Agwanda B, et al. Urban leptospirosis in Africa: a cross-sectional survey of Leptospira infection in rodents in the Kibera urban settlement, Nairobi, Kenya. The American journal of tropical medicine and hygiene. 2013;89(6):1095–102. Epub 2013/10/02. doi: 10.4269/ajtmh.13-0415. PubMed PMID: 24080637; PubMed Central PMCID: PMCPmc3854886.

48. Ahmed A, Engelberts MF, Boer KR, Ahmed N, Hartskeerl RA. Development and validation of a real-time PCR for detection of pathogenic leptospira species in clinical materials. PLoS One. 2009;4(9):e7093. Epub 2009/09/19. doi: 10.1371/journal.pone.0007093. PubMed PMID: 19763264; PubMed Central PMCID: PMC2740861.

49. Rahelinirina S, Leon A, Harstskeerl RA, Sertour N, Ahmed A, Raharimanana C, et al. First isolation and direct evidence for the existence of large small-mammal reservoirs of Leptospira sp. in Madagascar. PLoS One. 2010;5(11):e14111. Epub 2010/12/03. doi: 10.1371/journal.pone.0014111. PubMed PMID: 21124843; PubMed Central PMCID: PMC2991340.

50. Branger C, Blanchard B, Fillonneau C, Suard I, Aviat F, Chevallier B, et al. Polymerase chain reaction assay specific for pathogenic Leptospira based on the gene hap1 encoding the hemolysis‐associated protein‐1. FEMS Microbiol Lett. 2005;243(2):437–45.

51. Desvars A, Naze F, Benneveau A, Cardinale E, Michault A. Endemicity of leptospirosis in domestic and wild animal species from Reunion Island (Indian Ocean). Epidemiol Infect. 2013;1(1):1–12.

52. Houemenou G, Ahmed A, Libois R, Hartskeerl RA. Leptospira spp. Prevalence in Small Mammal Populations in Cotonou, Benin. ISRN Epidemiology. 2013;<http://dx.doi.org/10.5402/2013/502638>.

53. Jobbins S, Sanderson C, Alexander K. Leptospira interrogans at the Human-Wildlife Interface in Northern Botswana: A Newly Identified Public Health Threat. Zoonoses Public Health. 2013:10.111/zph.12052. Epub 14 May 2013. doi: 10.111/zph.12052.

54. Woo TH, Patel BK, Smythe LD, Symonds ML, Norris MA, Dohnt MF. Identification of pathogenic Leptospira genospecies by continuous monitoring of fluorogenic hybridization probes during rapid-cycle PCR. J Clin Microbiol. 1997;35(12):3140–46.

55. Lagadec E, Gomard Y, Guernier V, Dietrich M, Pascalis H, Temmam S, et al. Pathogenic Leptospira spp. in Bats, Madagascar and Union of the Comoros. Emerg Infect Dis. 2012;18(10):1696–97.

56. Smythe LD, Smith IL, Smith GA, Dohnt MF, Symonds ML, Barnett LJ, et al. A quantitative PCR (TaqMan) assay for pathogenic *Leptospira* spp. BMC Infect Dis. 2002;2(1):13–9.

57. Dietrich M, Wilkinson DA, Soarimalala V, Goodman SM, Dellagi K, Tortosa P. Diversification of an emerging pathogen in a biodiversity hotspot: Leptospira in endemic small mammals of Madagascar. Mol Ecol. 2014;23(11):2783–96. PubMed PMID: BIOSIS:PREV201400524773.

58. Nimo Paintsil SC, Fichet-Calvet E, Mohareb E, Morales M, Bonney JH, Obiri-Danso K, et al. Rodent species and their correlation with human seropositivity for zoonotic infections in Ghana. Am J Trop Med Hyg. 2013;89 (Suppl 1):422. PubMed PMID: 71313222.

59. Hatem ME, Ata NS, Abdou AM, Ibrahim ES, Bakry MA, Samir A. Surveillance of bovine leptospirosis: isolation and serodiagnosis. Global Veterinaria. 2014;13(1):127–32. PubMed PMID: CABI:20143243253.
